# Supplementary material for: Optimised fluorescence-activated nuclei sorting for epigenomic analysis of cortical cell types
Source: PLoS One. 2026 Oct 6;21(10):e0359643. doi: 10.1371/journal.pone.0359643 (PMC13641707; doi:10.1371/journal.pone.0359643)
Supplement: Supplementary file 2 [file pone.0359643.s002.pdf]

Sep 09, 2026 Version 4

Purification of human cortex excitatory neuron nuclei from fetal and postnatal tissue using fluorescent activated nuclei sorting (FANS) in combination with a SATB2 antibody. V.4

Version 1 is forked from Fluorescence-activated nuclei sorting (FANS) on human post-mortem cortex tissue enabling the isolation of distinct neural cell populations for multiple omic profiling

DOI
https://dx.doi.org/10.17504/protocols.io.n92ldz9d8v5b/v4

Jonathan P Davies[1], Stefania S S Policicchio[1], Barry Chioza[1], Gina Commin[1], Joe Burrage[1], Emma L Dempster[1], Jonathan Mill[1]

[1]University of Exeter Medical School, Exeter, UK

Complex Disease Epige...

Jonathan P Davies

Create & collaborate more with a free account

Edit and publish protocols, collaborate in communities, share insights through comments, and track progress with run records.

Create free account

OPEN ACCESS

DOI: https://dx.doi.org/10.17504/protocols.io.n92ldz9d8v5b/v4

Protocol Citation:
Jonathan P Davies, Stefania S S Policicchio, Barry Chioza, Gina Commin, Joe Burrage, Emma L Dempster, Jonathan Mill 2026. Purification of human cortex excitatory neuron nuclei from fetal and postnatal tissue using fluorescent activated nuclei sorting (FANS) in combination with a SATB2 antibody.. **protocols.io**
https://dx.doi.org/10.17504/protocols.io.n92ldz9d8v5b/v4
Version created by
Barry Chioza

License: This is an open access protocol distributed under the terms of the **Creative Commons Attribution License**, which permits unrestricted use, distribution, and reproduction in any medium, provided the original author and source are credited

Protocol status: Working
We use this protocol and it's working

Created: September 03, 2026

Last Modified: September 09, 2026

Protocol Integer ID: 324434

Keywords: FANS, post-mortem brain, nuclei, flow cytometry, anti-NeuN, nuclei sorting, anti-SATB2, fetal, neurodevelopment, FACS, neuron, development, nuclei from multiple different human brain cell, optimal marker for neuronal nuclei, purification of human cortex excitatory neuron nuclei, neuronal nuclei, nuclei from excitatory neuron, cellular composition in regulatory genomic study, multiple different human brain cell, human cortex excitatory neuron nuclei, heterogeneous mix of different neural cell type, epigenetic process, satb2 antibody, fetal cortex, different neural cell type, postnatal cortex, regulatory genomic study, open chromatin analysis, gene regulation, current analyses of the human brain, genome, human brain, postnatal tissue, rna, activated nuclei, specific patterns of gene regulation, transcriptional variation in health, specific cell, purified populations of nuclei, transcriptional variation, gene expression, cellular composition, determining cell type, dna, excitatory neuron, neuron, nuclei, cortex, cell type,

Funders Acknowledgements:
Alzheimer's Research UK (ARUK)
Grant ID: ARUK-PPG2018A-010
Simons Foundation for Autism Research (SFARI)
Grant ID: 573312
Medical Research Council UK (MRC UK)
Grant ID: MR/R005176/1

Abstract

Increased understanding of the functional complexity of the genome has led to growing recognition about the role of epigenetic/transcriptional variation in health and disease. Current analyses of the human brain, however, are limited by the use of “bulk” tissue, comprising a heterogeneous mix of different neural cell types. Because epigenetic processes play a critical role in determining cell type-specific patterns of gene regulation it is important to consider cellular composition in regulatory genomic studies of human post-mortem tissue, and there is a need for methods to purify populations of specific cell-types. This protocol builds on a **previous protocol** that uses fluorescence-activated nuclei sorting (FANS) to isolate and profile nuclei from multiple different human brain cell-types from frozen post-mortem tissue. Because NeuN is not an optimal marker for neuronal nuclei from fetal cortex, we have optimized a method using a SATB2 antibody to purify nuclei from excitatory neurons in both fetal and postnatal cortex. Purified populations of nuclei are amenable to simultaneous profiling of i) DNA modifications (via bisulfite sequencing / array), ii) histone modifications (via CUT&Tag), iii) open chromatin analysis (via ATAC-seq), and iv) gene expression (via RNA-seq).

Materials

	A	B	C
	**Equipment**	**Supplier**	**Catalogue No**
	**BD FACSAria™ III Cell Sorter**	BD Biosciences	648282-23
	**Sorvall WX 80+ Ultracentrifuge**	Thermo Scientific	75000080
	**7mL Dounce Tissue Grinder**	DWK Life Sciences	357542
	**PA Thin-walled ultracentrifuge tubes**	Thermo Scientific	10533934
	**Microcentrifuge Micro 17R - refrigerated**	Thermo Scientific	11526873
	**Tube Revolver Rotator**	Thermo Scientific	15238854

Table 1: Specifications of the equipment required for FANS protocol

	A	B	C
		**Supplier**	**Catalogue No**
	**D-Sucrose (Molecular Biology)**	Fisher Scientific	10638403
	**Calcium chloride (CaCl2) anhydrous, granular**	Sigma-Aldrich	C1016-100G
	**Magnesium acetate (Mg(Ace)2), 1M aq. soln**	Alfa Aesar	J60041
	**UltraPure 0.5M EDTA, pH 8.0**	Invitrogen	15575020
	**Thermo Scientific 1M Tris-HCl Buffer, pH 8.0**	Fisher Scientific	15568025
	**1,4-Dithiothreitol (DTT) - crystalline powder**	Sigma-Aldrich	3483-12-3
	**Triton X-100**	Sigma-Aldrich	T9284
	**UltraPure DNase/RNase-Free Distilled Water (ddH2O)**	Fisher Scientific	12060346
	**Bovine Serum Albumin (BSA)**	Sigma-Aldrich	A9647-500G
	**PBS Phosphate-Buffered Saline (10X) pH 7.4**	Fisher Scientific	10722497
	**Thermo Scientific™ RiboLock RNase Inhibitor (40 U/µL)**	Fisher Scientific	10389109
	**TRIzol LS Reagent**	Invitrogen	11588616
	**BAMBANKER serum free cell freezing medium**	BioCat GmbH	BB03-NP
	**BD FACSDiva CS&T Research Beads**	BD Biosciences	655051

	A	B	C
	**BD FACS Accudrop Beads**	BD Biosciences	345249
	**BD FACSFlow Sheath Fluid 20L**	BD Biosciences	342003
	**BD FACS Clean Solution**	BD Biosciences	15875858
	**BD FACS Rinse Solution**	BD Biosciences	340346

***Table 2* :** Specification of reagents required for FANS protocol

	A	B	C
	**Lysis Buffer (LB)**		
		**Stock**	**Amount**
	0.32M Sucrose		5.47 g
	5mM CaCl2	1M	250 µL
	3mM Mg(Ace)2	1M	150 µL
	0.1mM EDTA	0.5M	10 µL
	10mM Tris-HCl, pH 8	1M	500 µL
	1mM DTT	3M	17 µL
	0.1% Triton X-100		50 µL
	Optional: RiboLock RNase Inhibitor 0.2U/µL	*40U/ µL*	*5 µL / 1mL*
	Adjust with ddH2O to		50 mL
	**1.8M Sucrose Solution (SS)**		
	1.8M Sucrose		30.78 g
	3mM Mg(Ace)2	1M	150 µL
	1mM DTT	3M	17 µL
	10mM Tris-HCl, pH8	1M	500 µL
	Adjust with ddH2O to		50 mL

	A	B	C
	**5% BSA Solution (BB)**		
	BSA		200 mg
	1x PBS		4 mL
	**Staining Buffer (SB)**		
	0.5% BSA	5% BSA Solution (BB)	400 µL
	10X PBS		400 µL
	Optional: RiboLock RNase Inhibitor 0.2U/µL	*40U/ µL*	*5 µL / 1mL*
	Adjust with ddH2O to		4 mL

Table 3 : Recipes for buffers and solutions required

	A	B
	**Supplier**	Thermo Scientific™
	**Model**	Sorvall™ WX 80+
	**Rotor**	TH-641
	**Speed**	25,200 RPM / 108670.8 x g
	**Acceleration**	9
	**Deceleration**	5
	**Temperature**	4°C

Table 4: *Ultracentrifuge specification and conditions*

	A	B	C	D	E
	**Antibody**	**Preconjugated**	**Supplier**	**Cat No**	**Dilution**
	Hoechst 33342	--	Abcam	ab228551	1:500
	Anti-SATB2	to Alexa Fluor 488	Abcam	ab196316	1:1000
	Anti-NeuN	to Alexa Fluor488	Millipore	MAB377X	1:1000

Table 5: List of antibodies required for FANS protocol

Nuclear prep for FACS separation (using SATB2 and Hoechst)

1 The protocol below yields approximately 660,000 SATB2^{+ve}, 800,000 SATB2^{-ve} nuclei per 200 mg of frozen human fetal post-mortem cortex tissue. Recovery might vary from sample to sample due to high inter-sample variability (developmental stage and density of brain collection, cortex sub-areas, fat content of tissue sectioned, and white to grey matter ratio). NeuN yield is dependent on developmental age. While NeuN is not suitable for isolating nuclei from prenatal samples, it can be used in postnatal and adult cortex to recover a comparable population.

Refer to Materials-***Table 1*** for details about the equipment required and to Materials-***Table 2*** for specifications of reagents required.

Note

Throughout this protocol, SATB2 and NeuN are interchangeable as the neuronal antibody marker, depending on age of the individual being profiled.

1.1 ***Solution and buffer preps***

- *Lysis Buffer (LB)*
- *Sucrose Solution (SS)*
- *Staining Buffer (SB)*

Solutions should be kept at 4 °C or On ice. Refer to Materials-***Table 3*** for recipes of solutions and buffers.

Note

NOTE 1 - LB and SS can be prepared a week in advance, with DTT added on the day of use. Solutions should be stored at 4 °C once made, preferably in brown glass bottles.

Note

NOTE 2 - SB should be prepared fresh each day.
Concentration of BSA can be increased to up to 2% if nuclei "clumping" is encounted and excessive blocking of the nozzle occurs whilst sorting.

Note

NOTE 3 - Samples are homogenised as bulk tissue using a 7 mL Dounce homogeniser and then equally divided into two ultracentrifuge tubes.

1.2 ***Nuclei isolation*** 1h 20m

1. Pre-cool the ultracentrifuge, including the rotor and swing buckets, to 4 °C 00:30:00 before starting this stage of the protocol.
2. All buffers and the Dounce homogenisers should be pre-cooled on ice.
4. Add DTT ([M] 1 millimolar (mM) final concentration) to the SS and LB (i.e. 17 µL 3M DTT per 50 mL of SS/LB)
5. Transfer 2 mL LB to the homogeniser per 200 mg human brain tissue
6. Add the dissected tissue sample into the homogeniser
7. Wait 00:05:00 before douncing the tissue to allow the sample to defrost

To reduce heat caused by friction, the Dounce homogenisation step should be performed on ice with gentle strokes, and care should be taken to avoid foaming.

Note

NOTE 1 - Using the "TIGHT" pestle helps reduce the number of strokes required to reach full tissue disruption.

Note

NOTE 2- The number of strokes required to fully homogenise the tissue may vary between samples due to heterogeneity in cellular composition, lipid content, and the amount of connective tissue.

Note

NOTE 3- In comparison to adult tissue, the fetal tissue dissociates very easily, with fewer strokes required, and the lysate will be very pale. However, the cells are densely packed in a fetal brain, so we retained the same volume of lysis buffer to perform the dissociation and staining protocols.
Because the fetal brain is more easily dissociated, fewer strokes are required, and we have even seen success with gentle pipetting to agitate the tissue, rather than using a dounce.

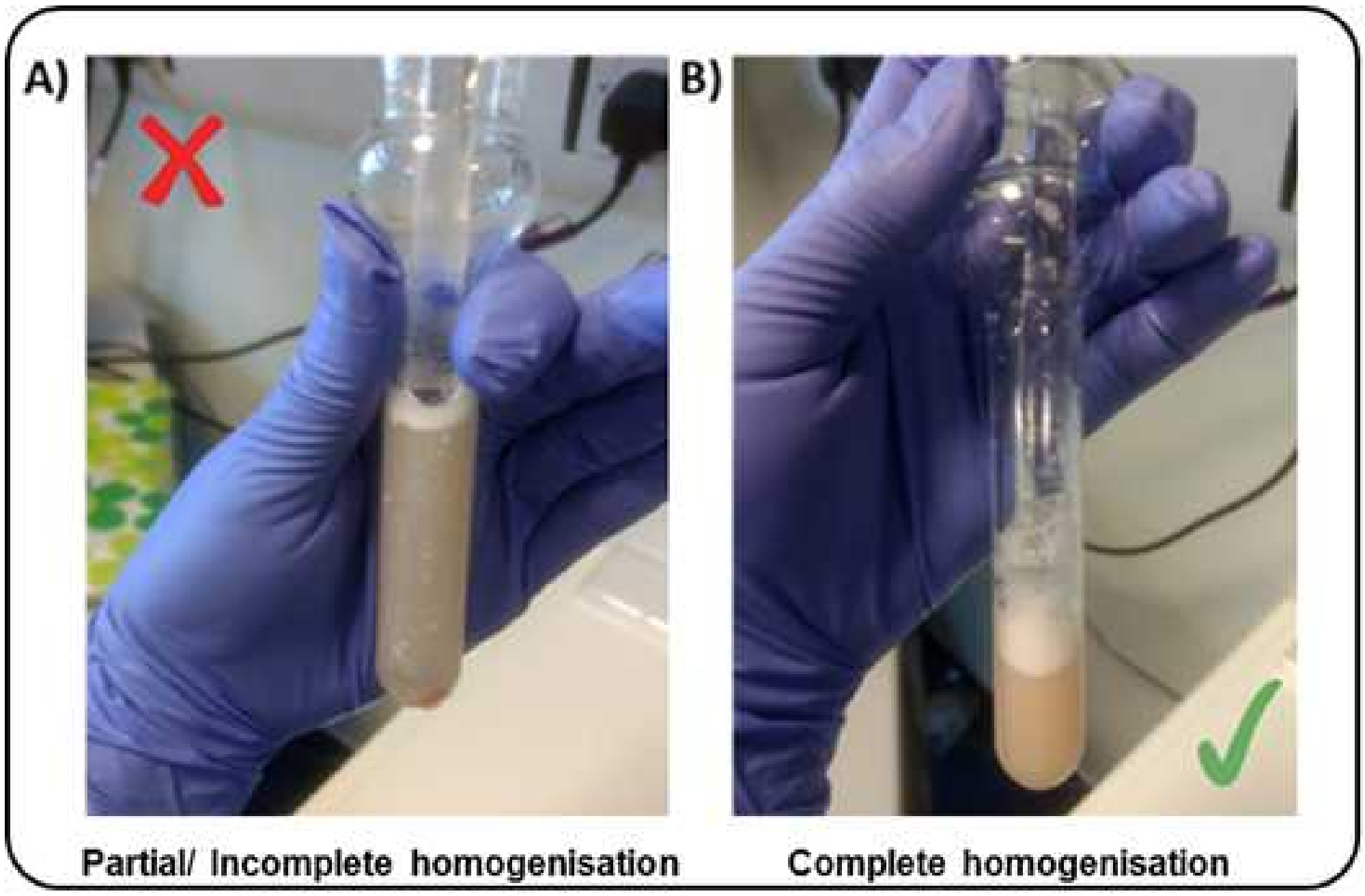

Figure 1 Example of adult brain tissue sample
A) only partially homogenised **B)** complete homogenisation.
Fetal samples will appear paler, with fewer pieces of floating lipid based tissue.

8.Transfer 8 mL SS (1.8M) to PA thin-walled ultracentrifuge tubes

9.Carefully overlay with tissue homogenate (1 mL per tube) - using a P1000 pipette, releasing slowly down the side of the tube

10.Overlay with another 1 mL LB – do not worry about disrupting the homogenate phase

11.Balance opposite tubes by weight with 1x PBS using a fine microbalance

12.Perform ultracentrifugation for 00:45:00 (see ***Table 4*** for centrifuge specification and conditions)

1.3 ***After Ultracentrifugation step***

1. Pour off the supernatant, taking care not to dislodge the pellet (90-degree inclination of the tube). If the pellet is hard to see, it is okay to leave 100-200 µL solution in the ultracentrifuge tube.
2. Re-suspend pellet in 750 µL SB and gently pipette up and down.
3. Let samples sit on ice for at least 00:15:00 , On ice

Blocking step

4. Transfer volume into 2 mL Lo-Bind DNA Eppendorf tubes.
5. Rinse out ultracentrifuge tubes to maximise nuclei collection by adding SB to each ultracentrifuge tube (1 mL) pipetting up and down several times and transferring to the new 2 mL tubes.

Washing step

6. Centrifuge tubes at 1000 x g for 00:05:00 , On ice
7. Discard supernatant (pipetting off, or pouring off gently).
8. Re-suspend each nuclei pellet in fresh SB (500 µL)
9. If the sample was split, then pool together pellets from the same sample (Final Volume = 1 mL)
10. Add DNA dye (Hoechst, 1:500) and mix thoroughly via inversion.
11. Pipette out 100 µL of nuclei solution for the Unstained Control (Hoechst dye only) and transfer to a new 2 mL tube.
12. Bring the volume up to 500 µL for the Unstained tube with fresh SB.
13. Replace the 100µL taken from the "Stained" tube with 100 µL of fresh SB (Final Volume = 1 mL)

Note

We don't add 1 mL of SB to the ultracentrifuge pellet, as there is always residual liquid remaining, and this would push the volumes above the 2 mL maximum in the 2 mL tubes.

Note

The amount of sample removed for the unstained control can vary, if you're concerned about wasting material. It is only used to establish the gating thresholds, or to collect a non-sorted population.

1.4 ***Immunostaining***

1. Add your antibody of choice (Ab) to staining tube:

SATB2 Alexa488 (1:1000)

or

NeuN Alexa488 (1:1000)

Refer to ***Table 5*** for specifications of the antibodies used

2. Incubate tubes for 01:30:00 on the rotator (speed=14 max) at 4 °C , keeping the tubes in the dark
3. Washing step: 1000 x g for 00:05:00 , 4 °C (both "Stained" and "Unstained" tubes)
4. Discard supernatant (by pipetting off)
5. Re-suspend in fresh SB (1 mL for the Unstained tube, 1.5-2 mL for the Stained tube - depending on pellet size)

Fluorescence-Activated Nuclei Sorting (FANS)

2 For machine start-up, CST and Accudrop calibrations refer to **BD FACSAria III User's Guide** for guidance and troubleshooting. The following instructions describe FANS using BD FACSAria III. Other FACS platforms can be used but might require modifications to the protocol.

2.1 ***General Gating Parameters***

For each sample, load stained and unstained tubes individually for data acquisition. A preliminary qualitative analysis of the acquired data is essential to select the appropriate gating strategy to maximize the nuclei capture while excluding unnecessary debris and to ensure optimal signal/noise ratio.

Gating Parameters (X-axis:Y-axis):

FSC-A:SSC-A (Size, cell granularity or internal complexity)

SSC-W:SSC-A (to gate out doublets)

FSC-A:DAPI-A (to gate the single nuclei population)

DAPI-A:FITC-A (to gate SATB2 or NeuN stained nuclei)

Note

Refer to Figure 2 for a visualisation of the gating strategy. Gating thresholds were kept as consistent as possible across developmental stages but were adjusted where necessary to account for developmental changes in marker expression and population distribution. Importantly,

the NeuN+ populations remain represented within the SATB2+ gate, allowing comparable population recovery when using SATB2.

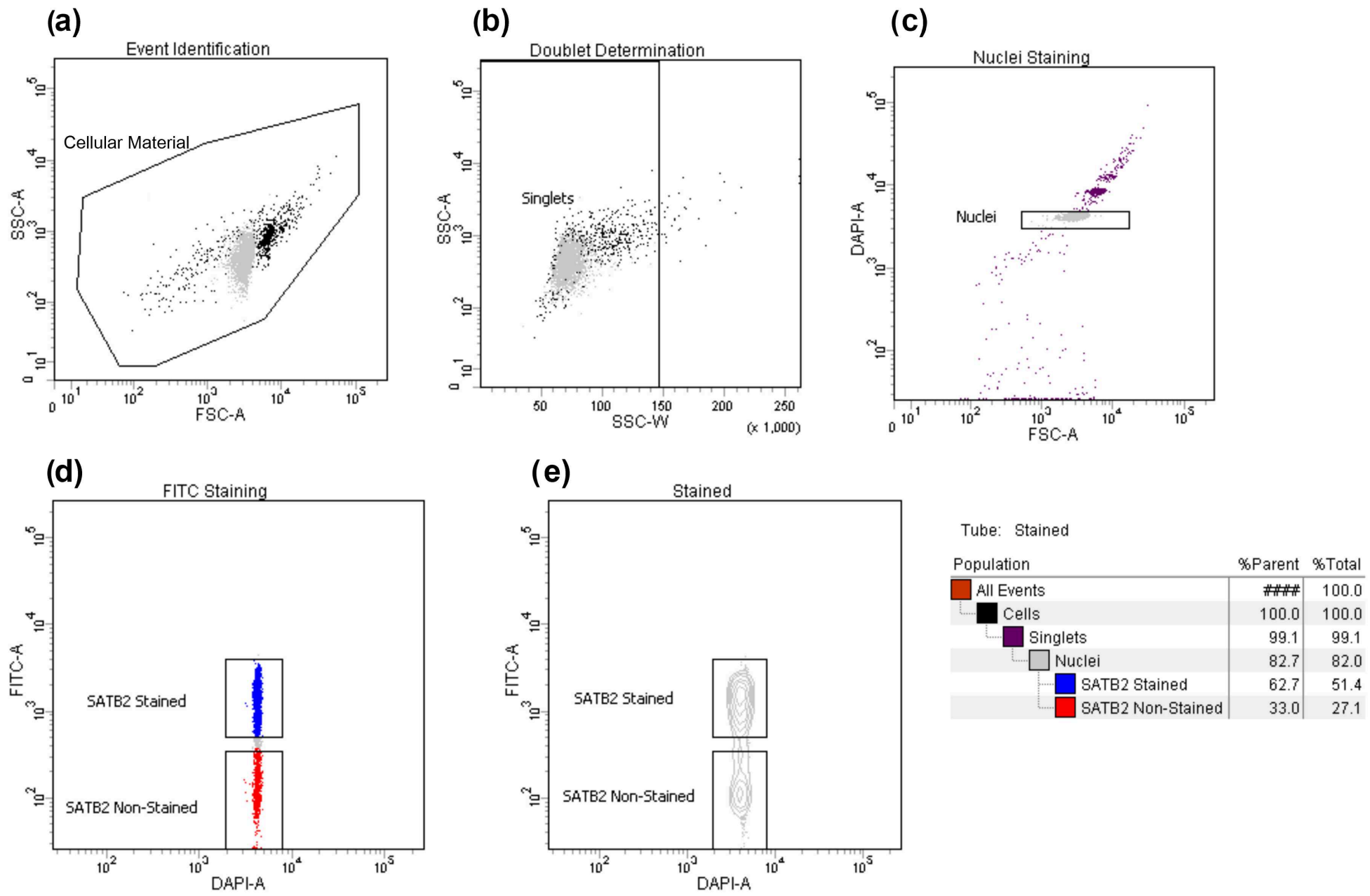

Figure 2. FANS gating strategy on a fetal sample 13 post conception weeks of development (13 pcw).
(a) Particles smaller than nuclei were eliminated with an area plot of forward-scatter (FSC-A) versus side-scatter (SSC-A), with gating for nuclei-sized particles inside the gate (box).
(b) Plots of width versus area in the side scatter channel are used for doublet discrimination with gating to exclude aggregates of two or more nuclei.
(c) Doublet discrimination gating was used to isolate nuclei determined by subgating on Hoechst 33342 fluorescence (DAPI).
Subsequent scatterplots discerning:
(d) SATB2-Alexa Fluor488–conjugated antibody staining (blue) for SATB2 stained neuronal cells and SATB2 non-stained nuclei (red) for non-neuronal cells
(e) SATB2-Alexa Fluor488–conjugated antibody staining in a contour plot.

2.2 *Data recording settings*

In line with the experiment design, FSC, SSC, DAPI, FITC, are the parameters for which voltage values may need to be slightly adjusted due to experiment/ inter-sample variability.

It is advisable to set the threshold value at 200 during data recording. Moreover, in the acquisition dashboard tab, we recommend setting **Events to Record** ≤3000, **Event to display** ≤1000 and **Flow Rate** = 1.0 (1,000 events per second) in order to increase the accuracy of signal detection.

The flow rate can be increased during sample collection to reduce the sort time (ideally max events per second =1,500 for a 100-micron nozzle). However, higher flow rates impact the data resolution and accuracy of event detection, and subsequent sorting of cellular fractions (see **BD FACSAria III User's Guide** for details).

During analysis, recorded data is displayed in plots, while gates are used to define populations of interest for selection. **Figure 3** shows a representative example of the

two most common outcomes we often observe.

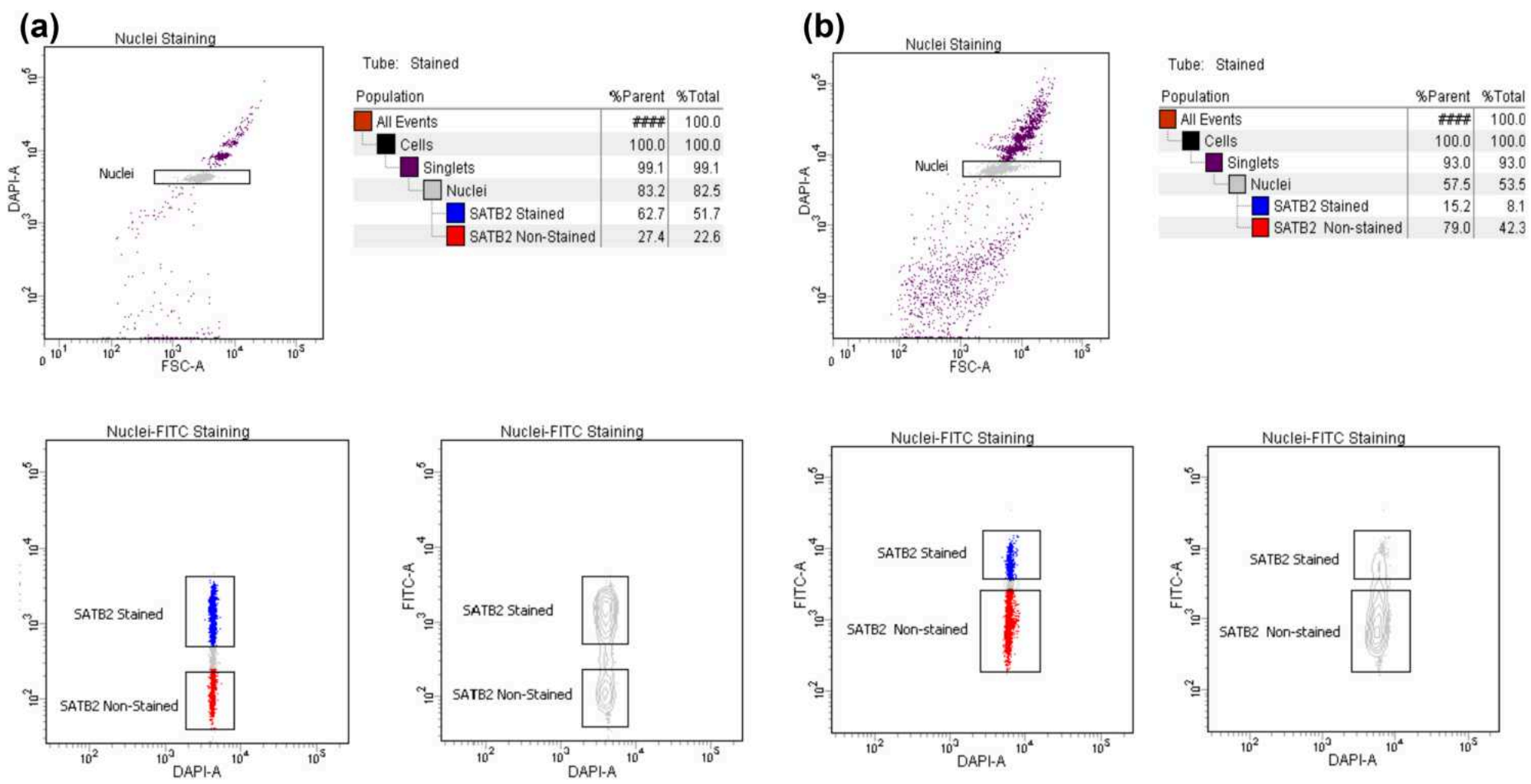

Figure 3. *Representative example of inter-individual variability.*

The data shown here are derived from two different control prefrontal cortex specimens of comparable age, gender, and brain collection which were processed in parallel following the same procedure.

a) sample from 13pcw, clean nuclei isolation, but with smaller SATB2^{+ve} fraction with clear separation of populations

b) Poor resolution between SATB2^{+ve} and SATB2^{-ve} populations in a 20 pcw sample; however, contour plots support the identification of distinct gates.

2.3 ***Sample Collection***

1. LoBind Tubes (Eppendorf, Cat No:30108051) are required to collect nuclei (to maximize sample recovery of nucleic acids by significantly reducing sample–to–surface binding).
2. Collected fractions can be used directly for downstream applications (e.g. DNA/RNA extraction, chromatin shearing).
3. If you are collecting for DNA or RNA, as soon as the number of events desired is reached, transfer the tubes On ice, do not hold them at Room temperature.

Note

During collection, it is crucial to regularly pause the sorting to mix the two phases in order to preserve the integrity of the resulting RNA preparations.

Note

For RNA extraction, LoBind Tubes should each contain 1 mL of pre-chilled TRIzol™ LS Reagent (Fisher Scientific, Cat No: 11578616) before sorting. If possible, use a chilled collection tube holder on your FACS machine.

4. Keep samples On ice and in the dark for the entire duration of the sorting.
5. Lightly vortex sample tubes to make the mixture homogeneous (not clumped) before loading the tube into the FACS chamber.
6. Load the **UNSTAINED** control tube into the chamber first to postion Unstained gating boxes.
7. Proceed by collecting **STAINED** tube by simultaneously sorting for SATB2^{+ve} (positive) or SATB2^{-ve} (negative) and proceed with nuclei collection.

Note

We observe considerable variability in staining profiles across fetal samples throughout development. Therefore, users should not expect the highly conserved staining patterns typically observed in adult cortex.

8. For long term storage of collected nuclei 1000 x g, 4°C for 00:05:00
9. Carefully remove supernatant.
10. Add 100 µL BAMBANKER to the tube.
11. Gently resuspend.
12. Store in -80 °C freezer

General Recommendations for the user

3 For every new experiment we recommend performing the following steps:
When loading your tube into the FACS machine, run the unstained / IgG control sample first as this aids in setting the baseline parameters
Check your **event rate** in the **Acquisition Dashboard** window. If it is greater than 1500 evt/s turn down the "**flow rate**" or unload and dilute the sample further. If less than 100 evt/s, turn up the "**flow rate**" (don't exceed a flow rate of 5.0 if possible, as the instrument is less focused and more inaccurate at higher flow rates)
In the **Acquisition Dashboard** window choose the appropriate "**stopping gate**" and "**storage gate**" (when working with nuclei, set as "Nuclei" and "All events" respectively)
Choose the range of "**events to record**" and "**events to display**" that best suits your purpose (≥ 5000 for both is advised)

Under the **"threshold"** tab in the Cytometer window, change the threshold (should be set for FSC) so that any small events in the bottom corner of the FSC vs SSC graph (caused by general cell debris and dust) are no longer shown. The threshold should not be set too high so that it causes an arbitrary, artificial cut-off through the left side of your population but not so low that small events caused by debris/dust are visible (ideally between a threshold 200-500).

Under the **"parameters"** tab in the Cytometer window, adjust the "**FSC**" and "**SSC"** values to get your population sitting in the centre of the FSC vs SSC graph (a re-adjustment of the "**threshold**" may be required at this point). It is essential to select **"restart**" each time any of the parameters are changed to update the events being displayed to ensure only events are recorded under the new settings.

Adjust or draw a new gate in the FSC vs SSC plot to encompass the population of interest.

Look in the scatter graph of SSC-A vs SSC-W (if you opened a blank experiment you will need to draw one). Right-click on the graph and check it is only displaying the events encompassed by your previous FSC vs SSC gate. Adjust or draw a gate for SSC A vs SSC W to encompass all of the main population to the left of the graph and exclude outliers to the right (these are doublets and other cell debris clumps)

Under the **"parameters"** tab in the Cytometer window adjust parameters for the fluorochromes selected so the unstained / IgG control sample sits close to 0 for the fluorochrome on a graph of FSC vs fluorochrome.

Load the stained samples and check the stained population has a clear increase in signal for the fluorochrome in comparison to the unstained (signal generally should not exceed 10^{x4}). Several minor re-adjustments of the fluorochrome's **"parameters"** may be necessary for the stained sample at this stage. If so, the unstained / IgG control has to be reset and re-recorded.

Note

WARNING– Do not alter acquisition parameters between samples intended for direct comparison. Any changes to acquisition settings will require all samples to be re-recorded using the updated parameters.

11. Select the correct option for the collection device in the **Sort Layout** window (we recommend "**4-Way Purity**" for general collection)

12. Regularly check your "**Efficiency**" in the **Sort Layout** window value. Between 80-100% is ideal, 70% is acceptable if less than 70% either the sample is too concentrated or you are sorting a rare population. Although the "**flow rate**" in the **Acquisition Dashboard** window can be increased to make the sort quicker, faster flow rates will negatively impact the efficiency.

13. Check the **"Electronic abort rate"** (N° errors /sec) and **"Electronic abort count"** (Tot N° of errors) at the bottom of the **Acquisition Dashboard** window. These parameters measure potential miss-sorts (different from efficiency as efficiency

measures undetermined drops which are directed to the “**Waste**” and therefore lost but do not contaminate).**“Electronic abort rate”** should be <1% of total events per second.
14. For long sorts, gate positions should be regularly monitored, especially for stained populations as fluorochromes lose intensity over time and the population can shift towards the unstained. Gates can be moved during long sorts to compensate.
