# Supplementary material for: Optimised fluorescence-activated nuclei sorting for epigenomic analysis of cortical cell types
Source: PLoS One. 2026 Oct 6;21(10):e0359643. doi: 10.1371/journal.pone.0359643 (PMC13641707; doi:10.1371/journal.pone.0359643)
Supplement: Supplementary file 3 [file pone.0359643.s003.pdf]

Sep 10, 2026 Version 5

Fluorescence-activated nuclei sorting (FANS) of purified neural cell populations from mouse cortex for multi-omic profiling V.5

Version 1 is forked from Fluorescence-activated nuclei sorting (FANS) on human post-mortem cortex tissue enabling the isolation of distinct neural cell populations for multiple omic profiling

DOI
https://dx.doi.org/10.17504/protocols.io.dm6gpbwndlzp/v5

Stefania S Policicchio[1], Isabel Castanho[1], Barry Chioza[1], Joe Burrage[1], Jonathan P Davies[1], Jonathan Mill[1], Emma L Dempster[1]

[1]University of Exeter Medical School, Exeter, UK

Complex Disease Epige...

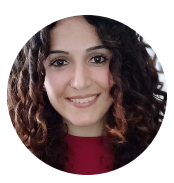

Stefania S Policicchio
University of Exeter Medical School

Create & collaborate more with a free account

Edit and publish protocols, collaborate in communities, share insights through comments, and track progress with run records.

Create free account

OPEN ACCESS

DOI: https://dx.doi.org/10.17504/protocols.io.dm6gpbwndlzp/v5

Protocol Citation:
Stefania S Policicchio, Isabel Castanho, Barry Chioza, Joe Burrage, Jonathan P Davies, Jonathan Mill, Emma L Dempster 2026. Fluorescence-activated nuclei sorting (FANS) of purified neural cell populations from mouse cortex for multi-omic profiling. **protocols.io**
https://dx.doi.org/10.17504/protocols.io.dm6gpbwndlzp/v5
Version created by
Barry Chioza

License: This is an open access protocol distributed under the terms of the **Creative Commons Attribution License**, which permits unrestricted use, distribution, and reproduction in any medium, provided the original author and source are credited

Protocol status: Working
We use this protocol and it's working

Created: September 10, 2026

Last Modified: September 10, 2026

Protocol Integer ID: 324711

Keywords: FANS, nuclei, flow cytometry, anti-NeuN, nuclei sorting, mouse cortex, anti- PU.1, nuclei from multiple different cell type, cellular composition in regulatory genomic study, purified neural cell population, other glial origin nuclei, frozen mouse cortex tissue, neural cell populations from mouse cortex, chromatin accessibility, epigenetic process, regulatory genomic study, purified populations of nuclei, heterogeneous tissue like the brain, different cortical cell type, microglia, frozen mouse cortex, specific patterns of gene regulation, gene regulation, multiple different cell type, genome, mouse cortex, histone modification, transcriptional variation in health, microarray, transcriptional variation, activated nuclei, determining cell type, dna modification, rna, populations of nuclei, dna, gene expression, cell type, cellular composition

Abstract

Increased understanding of the functional complexity of the genome has led to growing recognition about the role of epigenetic/transcriptional variation in health and disease. Because epigenetic processes play a critical role in determining cell type-specific patterns of gene regulation it is important to consider cellular composition in regulatory genomic studies of heterogeneous tissue like the brain. Building on a **previous protocol** for isolating purified populations of nuclei from different cortical cell types from human post-mortem brain tissue, this protocol uses fluorescence-activated nuclei sorting (FANS) to isolate and profile nuclei from multiple different cell types from frozen mouse cortex. This protocol can be used to robustly purify populations of neuronal (NeuN^{+ve}) and microglia (PU.1^{+ve}) and other glial origin nuclei (NeuN^{-ve}/PU.1^{-ve}) from frozen mouse cortex tissue, with each sample yielding purified populations of nuclei amenable to simultaneous analysis of i) DNA modifications (via bisulfite sequencing/microarray), ii) histone modifications, iii) chromatin accessibility (via ATAC-seq), and iv) gene expression (via RNA-seq).

Materials

	A	B	C
	**Equipment**	**Supplier**	**Catalogue No**
	**BD FACSAria™ III Cell Sorter**	BD Biosciences	648282-23
	**Sorvall WX 80+ Ultracentrifuge**	Thermo Scientific	75000080
	**1mL Dounce Tissue Grinder**	Sigma-Aldrich	DWK885300-0001-1EA
	**PA Thin-walled ultracentrifuge tubes**	Thermo Scientific	10533934
	**Microcentrifuge Micro 17R - refrigerated**	Thermo Scientific	11526873
	**Tube Revolver Rotator**	Thermo Scientific	15238854

Table 1: Specifications of the equipment required for FANS protocol

	A	B	C
		**Supplier**	**Catalogue No**
	**D-Sucrose (Molecular Biology)**	Fisher Scientific	10638403
	**Calcium chloride (CaCl2) anhydrous, granular**	Sigma-Aldrich	C1016-100G
	**Magnesium acetate (Mg(Ace)2), 1M aq. soln**	Alfa Aesar	J60041
	**UltraPure 0.5M EDTA, pH 8.0**	Invitrogen	15575020
	**Thermo Scientific 1M Tris-HCl Buffer, pH 8.0**	Fisher Scientific	15568025
	**1,4-Dithiothreitol (DTT) - crystalline powder**	Sigma-Aldrich	3483-12-3
	**Triton X-100**	Sigma-Aldrich	T9284
	**UltraPure DNase/RNase-Free Distilled Water (ddH2O)**	Fisher Scientific	12060346
	**Bovine Serum Albumin (BSA)**	Sigma-Aldrich	A9647-500G
	**PBS Phosphate-Buffered Saline (10X) pH 7.4**	Fisher Scientific	10722497
	**Thermo Scientific‰ RiboLock RNase Inhibitor (40 U/µL)**	Fisher Scientific	10389109
	**TRIzol LS Reagent**	Invitrogen	11588616
	**BAMBANKER serum free cell freezing medium**	BioCat GmbH	BB03-NP

	A	B	C
	**BD FACSDiva CS&T Research Beads**	BD Biosciences	655051
	**BD FACS Accudrop Beads**	BD Biosciences	345249
	**BD FACSFlow Sheath Fluid 20L**	BD Biosciences	342003
	**BD FACS Clean Solution**	BD Biosciences	15875858
	**BD FACS Rinse Solution**	BD Biosciences	340346

Table 2 : Specification of reagents required for FANS protocol

	A	B	C
	**Lysis Buffer (LB)**		
		**Stock**	**Amount**
	0.32M Sucrose		5.47 g
	5mM CaCl2	1M	250 µL
	3mM Mg(Ace)2	1M	150 µL
	0.1mM EDTA	0.5M	10 µL
	10mM Tris-HCl, pH 8	1M	500 µL
	1mM DTT	3M	17 µL
	0.1% Triton X-100		50 µL
	Optional: RiboLock RNase Inhibitor 0.2U/ µL	40U/ µL	5 µL / 1 mL
	Adjust with ddH2O to		50 mL
	**1.8M Sucrose Solution (SS)**		
	1.8M Sucrose		30.78 g
	3mM Mg(Ace)2	1M	150 µL
	1mM DTT	3M	17 µL
	10mM Tris-HCl, pH8	1M	500 µL
	Adjust with ddH2O to		50 mL

	A	B	C
	**5% BSA Solution (BB)**		
	BSA		200 mg
	1x PBS		4 mL
	**Staining Buffer (SB)**		
	0.5% BSA	5% BSA Solution (BB)	400 µL
	10X PBS		400 µL
	Optional: RiboLock RNase Inhibitor 0.2U/ µL	40U/ µL	5 µL / 1 mL
	Adjust with ddH2O to		4 mL

Table 3 : Recipes for buffers and solutions required

	A	B
	**Supplier**	Thermo Scientific™
	**Model**	Sorvall™ WX 80+
	**Rotor**	TH-641
	**Speed**	25,200 RPM / 108670.8 x g
	**Acceleration**	9
	**Deceleration**	5
	**Temperature**	4°C

Table 4 *:* Ultracentrifuge specifications and conditions

	Antibody	Preconjugated	Supplier	Cat No	Dilution
	Hoechst 33342	--	Abcam	ab228551	1:500
	Anti-NeuN	to Alexa Fluor488	Abcam	ab190195	1:1000
	Anti-PU.1	to PE	BioLegend	681308	1:100

Table 5 : List of antibodies used in FANS protocol

Protocol materials

BAMBANKER **BioCat GmbH Catalog #**BB03-NP

Nuclear prep for FACS separation (using NeuN, PU.1 and Hoechst)

1 In our hands, the protocol below yields ~60,000 $NeuN^{+ve}$ (neuron-enriched), ~5,000 $PU.1^{+ve}$ (microglial-enriched) and ~20,000 double-negative ($NeuN^{-ve}$/$PU.1^{-ve}$; oligodendrocyte-enriched) nuclei per ≤ 100 mg of frozen mouse cortex tissue. However, recovery can vary substantially between samples due to biological and technical factors, including developmental age, cortical region, dissection procedure, and tissue composition.

Refer to **Materials-Table 1** for details about the equipment required and to **Materials-Table 2** for specifications of reagents required.

1.1 ***Solution and buffer preps***

- *Lysis Buffer (LB)*
- *Sucrose Solution (SS)*
- *Staining Buffer (SB)*

Solutions should be kept at 4 °C or On ice . Refer to **Materials -Table 3** for recipes of solutions and buffers.

Note

NOTE 1 – LB and SS can be prepared a week in advance, with DTT added on the day of use. Solutions should be stored at 4 °C once made.

Note

NOTE 2 – SB should be prepared fresh each day.
Concentration of BSA can be increased to up to 2% if nuclei "clumping" is encounted and excessive blocking of the nozzle occurs whilst sorting.

Note

NOTE 3- Samples are homogenised as bulk tissue using a 1 mL Dounce homogeniser and then equally divided into two ultracentrifuge tubes.

1.2 ***Nuclei isolation*** 45m

1. Pre-cool the ultracentrifuge to 4 °C before starting this stage of the protocol.
2. All buffers and the Dounce homogenisers should be pre-cooled on ice.

3. Add DTT ([M] 1 millimolar (mM) final concentration) to the SS and LB (i.e. Amount 17 µL 3M DTT per 50 mL of SS/LB)
4. Transfer 1 mL LB into the homogeniser per 100 mg mouse brain tissue.
5. Add the dissected tissue sample into the homogeniser.
6. Wait 00:05:00 before douncing the tissue to allow the sample to defrost.

To reduce heat caused by friction, the Dounce homogenisation step should be performed on ice with gentle strokes, and care should be taken to avoid foaming.

Note

NOTE 1 – Using the "TIGHT" pestle helps reduce the number of strokes required to reach full tissue disruption.

Note

NOTE 2 - The number of strokes required to fully homogenise the tissue may vary between samples due to heterogeneity in cellular composition, lipid content, and the amount of connective tissue.

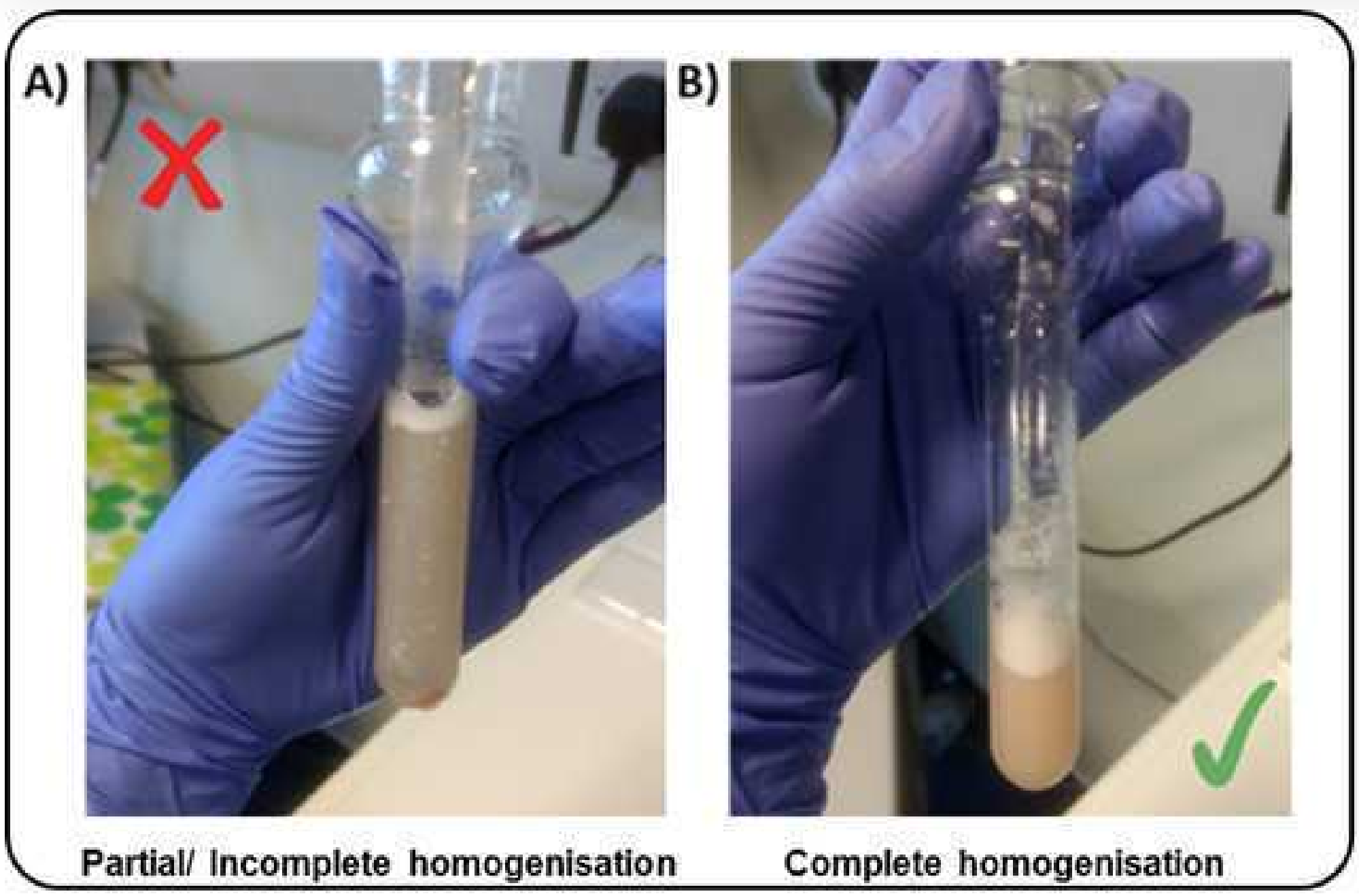

Figure 1 Example of brain tissue sample A) only partially homogenised B) complete homogenisation

7. Transfer 8 mL mL SS (1.8M) to PA thin-walled ultracentrifuge tubes.

8. Carefully overlay with tissue homogenate 1 mL per tube) - using a P1000 pipette, releasing slowly down the side of the tube.
9. Add 1 mL LB back into the homogeniser to rinse and recover as much as possible of the residual homogenate left behind.
10. Recover volume from homogeniser and overlay it on homogenate layer.
11. Balance opposite tubes by weight with 1x PBS using a fine microbalance.
13. Perform ultracentrifugation for 00:45:00 (see **Table 4** for centrifuge specification and conditions).

1.3 ***After Ultracentrifugation step*** 20m

1. Aspirate supernatant leaving 1-2 mL of the solution in the tube along with the pellet.
2. Pour off any remaining supernatant, taking care not to dislodge the pellet (90-degree inclination of the tube). If the pellet is hard to see, it is okay to leave 100-200 µL solution in the ultracentrifuge tube.
3. Resuspend pellet in 1 mL SB, gently pipette up and down.
4. Let samples sit On ice for at least 00:15:00

Blocking step

5. Transfer volume into a new 1.5 mL Lo-Bind DNA Eppendorf tube.
6. Rinse out ultracentrifuge tubes in order to maximise nuclei collection by adding 1 mL of SB per tube, pipetting up and down several times, and transferring into a new 1.5 mL LoBind DNA tube.

Washing step

7. Centrifuge tubes at 1000 x g for 00:05:00 , 4 °C
8. Discard supernatant (pipetting off gently).
9. Re-suspend each nuclei pellet in fresh SB (500 µL).
10. If the sample was split then pool together resuspended pellets from the same sample (Final Volume = 1 mL)
11. Add DNA dye (Hoechst, 1:500) and mix thoroughly by inversion.
12. Pipette out 120 µL of nuclei solution for the Unstained Control (Hoechst dye only) and transfer to a new 1.5 mL LoBind DNA tube.
13. Bring the volume up to 500 µL for the Unstained tube with fresh SB.
14. Replace the volume taken from the "Stained" tube with 120 µL of fresh SB (Final Volume = 1 mL).

1.4 ***Immunostaining*** 1h 35m

1. Add the following three antibodies (Ab) to the "Stained" tube (1mL final volume):

- PE anti-PU.1 (1:100 dilution) – [10 µL Ab]

- Alexa488 anti-NeuN (1:1000 dilution) – [1 µL Ab]

Refer to **Table 5** for specifications of the antibodies used

2. Incubate tubes for 01:30:00 on the rotator (speed=11) at 4 °C , keeping the tubes in the dark.
3. **Washing step**: Centrifugation at 1000 x g, 4°C for 00:05:00 (both "Stained" and "Unstained" tubes).
4. Discard supernatant (by aspiration).
5. Re-suspend in fresh SB (500 µL for the Unstained tube, 1 mL for the Stained tube - depending on pellet size).

Fluorescence-Activated Nuclei Sorting (FANS)

2 For machine start-up, CST and Accudrop calibrations refer to **BD FACSAria III User's Guide** for guidance and troubleshooting. The following instructions describe FANS using BD FACSAria III. Other FACS platforms can be used but might require modifications to the protocol.

2.1 ***General Gating Parameters***

For each sample, load stained and unstained tubes individually for data acquisition. A preliminary qualitative analysis of the data acquired is essential to select the appropriate gating strategy to maximise the nuclei capture while excluding unnecessary debris and to ensure an optimal signal/noise ratio.

Gating Parameters (X-axis: Y-axis):

FSC-A:SSC-A (Size, cell granularity or internal complexity)

SSC-W:SSC-A (to gate out doublets)

FSC-A:DAPI-A (to gate the single nuclei population)

DAPI-A:FITC-A (to gate NeuN stained nuclei)

DAPI-A:PE-A (to gate PU.1 stained nuclei)

FITC-A: PE-A (to visualize the distribution of the three detectable populations)

> Note
>
> The PU.1+ve population (microglia) is gated as a "daughter" population from the NeuN-ve fraction (non neuronal nuclei). Refer to **Figure 2** for a visualization of the gating strategy.

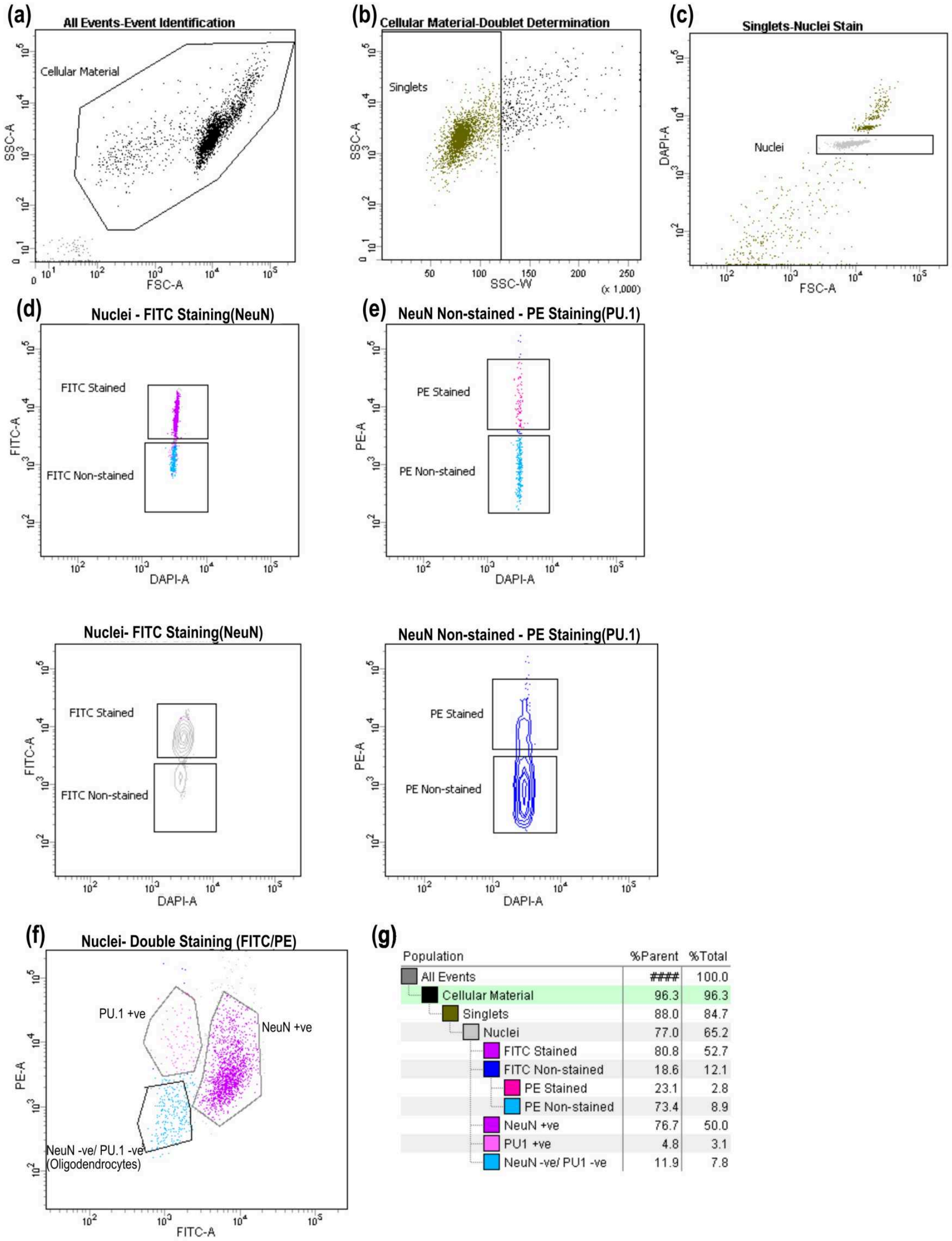

Figure 2. FANS gating strategy. **(a)** Particles smaller than nuclei were eliminated with an area plot of forward-scatter (FSC-A) versus side-scatter (SSC-A), with gating for nuclei-sized particles inside the gate (box). **(b)** Plots of width versus area in the side scatter channel are used for doublet discrimination with gating to exclude aggregates of two or more nuclei. **(c)** Doublet discrimination gating was used to isolate nuclei determined by sub-gating on Hoechst 33342 fluorescence (DAPI). f) Subsequent scatterplots discerning **(d)** NeuN-Alexa Fluor488–conjugated antibody staining (purple) **(e)** PU.1 PE-stained nuclei (dark pink) **(f)** the distribution of the three main nuclei subpopulations identified through double staining strategy ($NeuN^{+ve}$, neurons; $PU.1^{+ve}$, microglia, $double^{-ve}$, oligodendrocytes enriched). **(g)** The resultant hierarchical colour key ensures that only nuclei that are negative for staining with the NeuN antibody are passed through the next gating condition.

2.2 ***Data recording settings***

In line with the experiment design, FSC, SSC, DAPI, FITC and PE are the parameters for which voltage values may need to be slightly adjusted due to experiment/ inter-sample variability.

It is advisable to set the threshold value between 200 and 500 during data recording. Moreover, in the acquisition dashboard tab, we recommend setting **Events to Record**≤3,000, **Event to display**≤5000 and **Flow Rate** = 1.0 (1,000 events per second) in order to increase the accuracy of signal detection.

The flow rate can be increased during sample collection to reduce the sort time (ideally max events per second =1,500 for a 100-micron nozzle). However higher flow rates impact the data resolution and accuracy of events detection, and subsequent sorting of cellular fractions (see BD FACSAria III User's Guide for details).

During analysis, recorded data is displayed in plots, while gates are used to define populations of interest for selection. **Figure 3** shows a representative example of the two most common outcomes we often observe.

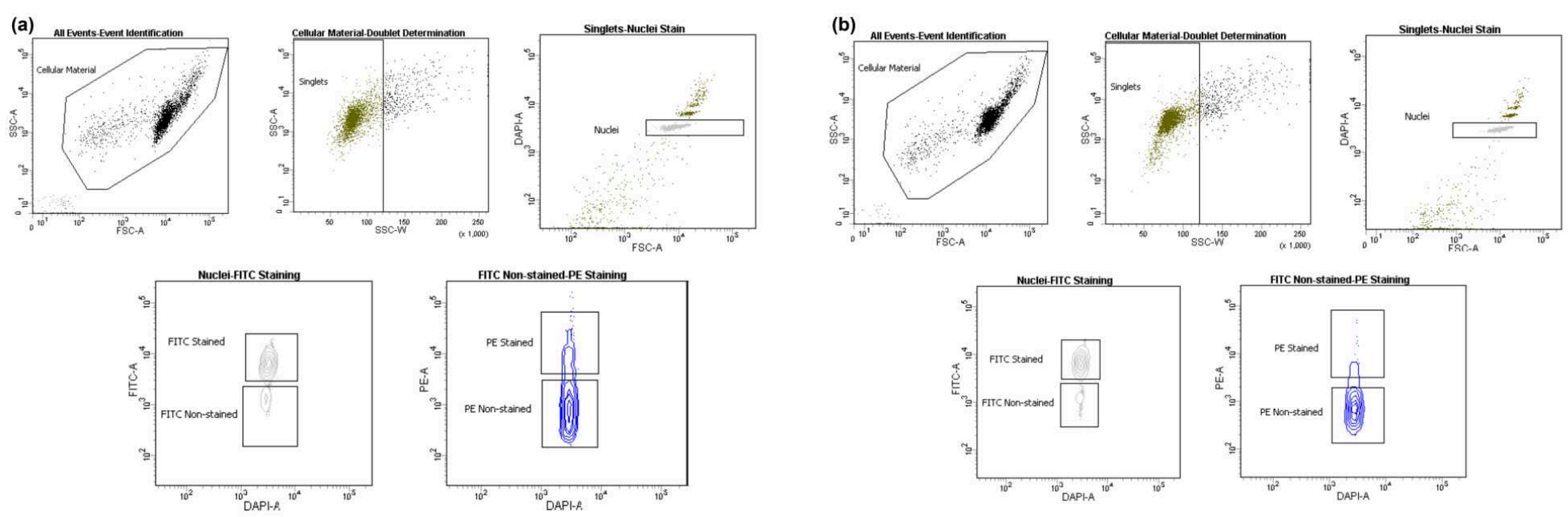

Figure 3. Representative example of inter-individual variability. The data shown here are derived from two different cortex specimens of comparable age, sex, and group which were processed in parallel following the same procedure.
(a) distinctly separated PU.1 +ve fraction vs **(b)** missing PU.1+ve positively stained population.

2.3 **Sample Collection**

1. LoBind Tubes (Eppendorf, Cat No:30108051) are required to collect nuclei (to maximise sample recovery of nucleic acids by significantly reducing sample–to–surface binding).
2. Collected fractions can be used directly for downstream applications (e.g. DNA/RNA extraction, ATAC-seq, etc) or stored in -80 °C freezer.
3. If you are collecting for DNA or RNA, as soon as the number of events desired is reached, transfer the tubes On ice , do not hold them at Room temperature

Note

During collection, it is crucial to regularly pause the sorting to mix the two phases in order to preserve the integrity of resulting RNA preparations. For RNA extraction, LoBind Tubes should each contain 500 µL of pre-chilled TRIzol LS **Fisher Scientific Catalog** #11588616 prior to sorting.

4. Keep samples On ice for the entire duration of the sorting.
5. Lightly vortex sample tubes to make the mixture homogeneous (not clumped) before loading the tube into the FACS chamber.
6. Load the **UNSTAINED** control tube into the chamber first and proceed with nuclei collection (for DNA, 200,000 events; for RNA, 300,000 events; for ATAC-seq 50,000 events).
7. Proceed by collecting If you are collecting for DNA or RNA, as soon as the number of events desired is reached, transfer the tubes On ice , do not hold them at Room temperature tube by simultaneously sorting for NeuN and PU.1

Note

The PU.1+ve population may not be detectable in every brain sample processed (high inter-individual variability); when it is, it represents between 5-10% of the total sample, therefore yielding often insufficient material for multiple assays.

8. For long term storage of collected nuclei 1000 x g , 4 °C for 00:05:00
9. Carefully remove supernatant.
10. Add 100 µL BAMBANKER **BioCat GmbH Catalog** #BB03-NP per 100,000 nuclei collected to the tube.
11. Gently resuspend.
12. Store in -80 °C freezer.

General Recommendations for the user

3

For every new experiment we recommend performing the following steps:

1. When loading your tube into the FACS machine, run the unstained / IgG control sample first as this aids in setting the baseline parameters
2. Check your **event rate** in the **Acquisition Dashboard** window. If it is greater than 1500 evt/s turn down the “**flow rate**” or unload and dilute the sample further. If less than

100 evt/s, turn up the "**flow rate**" (don't exceed a flow rate of 5.0 if possible, as the instrument is less focused and more inaccurate at higher flow rates)

3. In the **Acquisition Dashboard** window choose the appropriate "**stopping gate**" and "**storage gate**" (when working with nuclei, set as "Nuclei" and "All events" respectively)
4. Choose the range of "**events to record**" and "**events to display**" that best suits your purpose (≥ 5000 for both is advised)
5. Under the "**threshold**" tab in the Cytometer window, change the threshold (should be set for FSC) so that any small events in the bottom corner of the FSC vs SSC graph (caused by general cell debris and dust) are no longer shown. The threshold should not be set too high so that it causes an arbitrary, artificial cut-off through the left side of your population but not so low that small events caused by debris/dust are visible (ideally between a threshold 200-500).
6. Under the "**parameters**" tab in the Cytometer window, adjust the "**FSC**" and "**SSC**" values to get your population sitting in the centre of the FSC vs SSC graph (a re-adjustment of the "**threshold**" may be required at this point). It is essential to select "**restart**" each time any of the parameters are changed to update the events being displayed to ensure only events are recorded under the new settings.
7. Adjust or draw a new gate in the FSC vs SSC plot to encompass the population of interest.
8. Look in the scatter graph of SSC-A vs SSC-W (if you opened a blank experiment you will need to draw one). Right-click on the graph and check it is only displaying the events encompassed by your previous FSC vs SSC gate. Adjust or draw a gate for SSC-A vs SSC-W to encompass all the main population to the left of the graph and exclude outliers to the right (these are doublets and other cell debris clumps)
9. Under the "**parameters**" tab in the Cytometer window adjust parameters for the fluorochromes selected so the unstained / IgG control sample sits close to 0 for the fluorochrome on a graph of FSC vs fluorochrome.
10. Load the stained samples and check the stained population has a clear increase in signal for the fluorochrome in comparison to the unstained (signal should not exceed 10^4). Several minor re-adjustments of the fluorochrome's "**parameters**" may be necessary for the stained sample at this stage. If so, the unstained / IgG control must be reset and re-recorded.

Note

WARNING – Do not alter acquisition parameters between samples intended for direct comparison. Any changes to acquisition settings will require all samples to be re-recorded using the updated parameters.

11. Select the correct option for the collection device in the **Sort Layout** window (we recommend "**4-Way Purity**" for general collection)

12. Regularly check your "**Efficiency**" in the Sort Layout window value. Between 80-100% is ideal, 70% is acceptable if less than 70% either the sample is too concentrated or you are sorting a rare population. Although the "**flow rate**" in the **Acquisition Dashboard** window can be increased to make the sort quicker, faster flow rates are less efficient.
13. Check the "**Electronic abort rate**" (N° errors /sec) and "**Electronic abort count**" (Tot N° of errors) at the bottom of the **Acquisition Dashboard** window. These parameters measure potential miss-sorts (different from efficiency as efficiency measures undetermined drops which are directed to the "Waste" and therefore lost but do not contaminate)."**Electronic abort rate**" should be <1% of total events per second.
14. For long sorts, gate positions should be regularly monitored, especially for stained populations as fluorochromes lose intensity over time and the population can shift towards the unstained. Gates can be moved during long sorts to compensate.
