# Supplementary material for: Optimised fluorescence-activated nuclei sorting for epigenomic analysis of cortical cell types
Source: PLoS One. 2026 Oct 6;21(10):e0359643. doi: 10.1371/journal.pone.0359643 (PMC13641707; doi:10.1371/journal.pone.0359643)
Supplement: Supplementary file 5 [file pone.0359643.s023.docx]

Dr Emma Dempster FHEA

Associate Professor (Research & Education)

Complex Disease Epigenetics Group

University of Exeter Medical School
RILD Building South, Royal Devon & Exeter Hospital

Barrack Road, Exeter, EX2 5DW

Direct Line: +44 (0)7988726781

Email: [*e.l.dempster@exeter.ac.uk*](mailto:e.l.dempster@exeter.ac.uk)

19^th^ June 2026

**PLOS ONE Decision: Revision required [PONE-D-26-02584] -** [Optimised fluorescence-activated nuclei sorting for epigenomic analysis of cortical cell types]

Dear Prof. Nafisa M. Jadavji,

Thank you for the opportunity to revise our manuscript, “*Optimised fluorescence-activated nuclei sorting for epigenomic analysis of cortical cell types*”, for consideration in PLoS ONE.

We have carefully addressed all comments raised by you and the reviewer and have revised the manuscript in accordance with PLOS ONE style guidelines.

We are grateful for the reviewer’s positive and constructive evaluation of our work. In particular, we were encouraged by their recognition that “...*compared to other protocols these protocols describe optimization of parallel multi-omic profiling across multiple neural populations isolated from a small amount of post-mortem tissue*...”, and that the protocols “...w*ill be helpful to those investigating epigenomic changes in various cell types associated with disease using mouse and human tissue and make efficient use of tissue for data collection from multiple cell types*...”.

We have carefully considered all comments and have provided a detailed point-by-point response below. We believe that the revisions have strengthened the manuscript and improved its clarity while preserving the central methodological advances and practical utility of the protocol.

Further, for clarification, the funders had no role in study design, data collection and analysis, the decision to publish, or the preparation of the manuscript.

We hope that the revised manuscript is now suitable for publication in PLOS ONE and thank you again for your consideration.

Yours sincerely,

Dr Emma Dempster
On behalf of all authors

**1. Journal requirements**:

1. Please ensure that your manuscript meets PLOS ONE's style requirements, including those for file naming.

*We have consulted the templates and made the necessary alterations to ensure that our manuscript meets the style requirements.*

2. To comply with PLOS One submissions requirements, in your Methods section, please provide additional information regarding the experiments involving animals and ensure you have included details on (1) methods of sacrifice, (2) methods of anesthesia and/or analgesia, and (3) efforts to alleviate suffering.

*The Methods section has been revised to include additional details regarding animal welfare considerations. All animals were humanely euthanised by cervical dislocation performed by trained personnel in accordance with Schedule 1 of the UK Animals (Scientific Procedures) Act 1986. No anaesthesia or analgesia was administered before euthanasia, as cervical dislocation is an approved Schedule 1 method of humane killing and was considered appropriate for the procedures undertaken. Animals were monitored regularly throughout the study, maintained under standard husbandry conditions, and all efforts were made to minimise distress and suffering. These details have now been added to the Methods section of the revised manuscript.*

3. Please include your tables as part of your main manuscript and remove the individual files. Please note that supplementary tables should remain uploaded as separate "supporting information" files.

*We have embedded Table 1 into the main document as requested.*

4. Please include captions for your Supporting Information files at the end of your manuscript, and update any in-text citations to match accordingly. Please see our Supporting Information guidelines for more information: <http://journals.plos.org/plosone/s/supporting-information>.

*This has been completed as requested – please see revised files.*

**2. Additional Editor Comments**:


My comments to you are to describe whether there is a requirement for starting material quality. I think more trouble shooting tips should be included as well as representative results.

*Thank you for these comments. We have added more troubleshooting tips on protocols.io where a detailed step-by-step protocol is available, including comments regarding starting material quality and advice on how to deal with nuclei clumping.*

**3.Reviewers' comments:**

**Major suggestions for manuscript:**
1. Line 101: Use the PLoSOne Protocol template preferred language.

*We have used the preferred language template to reference our step-by-step protocol on protocol.io.*

2. General comment: In general, the flow cytometry data throughout S1-S3 files/manuscript are describing populations as positive and negative; however, without an unstained/IgG control to validate, it looks like in some cases, you might have high and low populations rather than a truly negative population. Could one example be included of an unstained control with the overlaid positively stained sample for an example of proper troubleshooting for a new marker?

*We thank the reviewer for this helpful suggestion and have included unstained images for each of our FANS protocols in the revised Supplementary Figures S1-S3. These additions provide a clear reference for establishing fluorescence thresholds and demonstrate the distinction between background autofluorescence and true antibody-positive populations. We hope this will aid users in troubleshooting and adapting the protocols to additional markers or tissue sources, while also addressing the reviewer's point that some populations may otherwise be interpreted as high- and low-expressing populations rather than truly negative and positive populations.*

3. Figure S7 and associated bulk RNA seq analysis: Do PC plots show clear separation of the distinct populations as is depicted by the clustering using the top variably expressed genes? Throughout, the cell type separation is validated by selection of the most variable genes/locations. It would be nice to have at least one PC analysis.

*We thank the reviewer for this helpful suggestion and agree that an orthogonal dimensionality reduction approach provides additional support for the separation of the isolated cell populations beyond clustering based on highly variable genes. To address this point, we have now included a principal component analysis (PCA) plot in Figure S7C generated using the 5,000 most variably expressed genes. The PCA demonstrates clear separation between the two cell populations, providing an independent validation of the clustering results shown previously. We have updated Figure S7 and the corresponding text in the manuscript to highlight this additional validation of cell-type separation.*

**Minor Suggestions for manuscript:**
1. Line 203: It mentions that two nuclei fractions were inspected, but specifies three (NeuN+, SOX10+ and total) populations. Please clarify if two or three and which populations were inspected.

*Thank you for spotting this. We have corrected it to state “three”.*

2. Number mismatch for DNA methylation (ONT). It’s listed that genomic DNA was harvested from 50K-200K nuclei in Figure 1, but line 236 lists that genomic DNA was extracted from 500,000 nuclei for ONT sequencing and line 314 mentions 50K. Please double check nuclei amounts used for ONT or if there is a description missing.

*Thank you for highlighting this inconsistency. The different DNA methylation profiling approaches used in this study required different input amounts of genomic DNA and, consequently, different numbers of nuclei. We have revised Figure 1 to accurately reflect the nuclei input requirements for each method and have updated the relevant sections of the text to clarify which nuclei numbers correspond to each DNA methylation profiling approach.*

3. Figure S9a; the labelling for DGFRB is covered/shortened.

*The PDGFRB label was inadvertently obscured by a text box in the figure. This has now been corrected in the revised Figure S9A.*

4. One of the strengths of this protocol is that it gives a good depiction of how much data can be gathered from 300mg of human cortex tissue, which greatly helps in planning such studies. For mouse tissue, far less cells are capable of being isolated (lines 317-319). Do the authors have a good sense of the use of the number of nuclei needed in multiomics approaches and how best to plan use of fewer nuclei across multiple analyses with mice? Any insight would be valuable to those researchers using mice.

*We thank the reviewer for this valuable suggestion and agree that practical guidance around experimental planning is particularly useful for researchers working with mouse tissue, where nuclei yields are often substantially lower than those obtained from human cortex.*

*The number of nuclei required depends on the downstream application and the abundance of the target cell population. Based on our experience, for mouse cortex it is generally feasible to generate both DNA methylation data using the mouse DNA methylation array and ATAC-seq data from the more abundant NeuN+ neuronal population, with approximately 10,000–30,000 nuclei allocated to each assay. However, yields can decrease considerably when working with smaller brain regions or rarer cell populations, making it challenging to perform multiple downstream assays from a single sample. In such cases, careful experimental prioritisation is essential. Researchers may need to optimise nuclei isolation procedures, pool tissue samples where appropriate, or prioritise assays according to study objectives and expected effect sizes. We have added text to both the methods section and to the discussion to provide guidance on expected nuclei requirements and considerations for experimental planning in mouse studies.*

5. Line 471 suggests that a limitation is the cytometry platform and fluorophore capabilities. While this can be a limitation as multicolor experiments become more complicated and need additional troubleshooting, the current FANS protocol only uses 3 markers (and Hoescht), for a total of 4 colors. This is not the full capacity of the Aria III instrument they used. Is it really the cytometer that is limited or is more that the markers are limited in the available antibodies with flow cytometry-appropriate fluorophores? Please clarify.

*Although the FACS Aira III can support up to 5 lasers, our model is a 4-laser-configured instrument, and our approach utilises the spatial separation of the 4 laser spots to avoid fluorochrome leakage and negate the need for compensation. While it could be possible to add additional markers as each laser has multiple detectors, this would involve a more complicated experimental setup requiring compensation for fluorochromes detected using a shared excitatory laser, which could potentially reduce the sensitivity of detection. For this reason, we utilised a 4-colour approach, although we acknowledge that more markers could potentially be added using a more complex experimental design and make this point in the manuscript.*

6. Grammar/language comments:
a. Line 82: run-on sentence; Add a colon after ‘downstream genomic assays’.

*Resolved.*

b. Line 199, 264, 443: space before or reference

*Resolved.*

c. Line 203: parenthesis after total is superscript

*Resolved.*

d. Line 358: double wording ‘validates, confirms or both’

*Resolved.*

e. Line 388: Insert comma, ‘postnatal human, please refer to’

*Resolved.*

f. Line 447: comma inserted ‘and other cell types (46), our protocols are’

*Resolved.*

g. Line 457: comma insertion/ letter case change: ‘and astrocytes (LHX2), highlight that this strategy’
*Resolved.*

**Minor suggestions to protocol S1-S3 files:**

*We thank the reviewer for these detailed corrections below and have amended the three protocols accordingly and created revised versions on Protocols.io.*

Protocol/ File S1:
1. Equipment missing: centrifuge, rotating mixer (File S1-S3).
2. Section 1.4, step 5: Suggested to resuspend in ‘-2mL’. Correct the negative volume.
3. Section 2.2 It’s mentioned that the flow rate can be increased in reduce sort ‘speed’. Are you actually referring to sort time, as increasing flow rate would increase the sort speed.
4. What is the interpretation for Figure 2: the FITC-A vs PE-A plot, it appears that NeuN+ cells are both PE+ and FITC+? Was compensation performed to address spectral overlap?
5. Grammar/language comments:
a. Section 2.3: Combine notes 1+2 about RNA Trizol and mixing tube so it’s clear what sample needs to be mixed. This happens again in File S2.
b. Section 2.3: Note 3: Repeated ‘at least’ for yield. Remove one.

Protocol/ File S2:
1. Section 1.3, step 6; no speed mentioned for centrifuge step.
2. Section 1.3, step 13: 1mL is repeated as final volume.
3. Section 1.4, step 2: instead of rotor, use rotator or rotating mixer.
4. There is a time for section 2, FANS as 5 minutes. This is misleading, as all the steps mentioned to start up the instrument and gating take much longer. Just remove a time as that would depend on sample.
5. Section 2 Note 1: There’s a reference made to ‘populations’ that should be present. What populations are being referred to here?
6. Figure 2 makes reference to letters for panels, but the panels are not labeled in the actual figure.
7. Section 2.2 mentions sort speed as reduced with a flow rate increase. See comment 3 under Protocol S1.
8. Figure 3b mentions poor sample separation. I suggest being more specific in that it is the population separation (Satb2+ versus Stab2-) that is poorer in this sample.
9. Grammar/language comments:
a. Section 1.3, step 1: ‘pour off any remaining supernatant’
b. There’s some strange spacing errors for section 1.3, step 2,3,9,12.
c. Section 1.3, step 9: insert comma, ‘If the sample was split, then pool together..’
d. Section 1.3, step 13: missing quotation around “Stained”
e. Section 2.3, numbers of steps are all repeated.
f. Note/Warning Section 3: Separate into two sentences instead of separating with comma.

Protocol/File S3:
1. Antibody and dyes (Table 5) are missing for this protocol file. Note that Table 5 is referenced in immunostaining step, section 1.4.
2. Section 1.2, step 5: Is there a suggestion for how much volume should be used per mg of tissue?
3. Section 1.3, step 7: Make clear that washing step instruction is a centrifugation.
4. Section 1.4, step 2: As with other files, use rotator or rotating mixer to explain equipment.
5. Grammar/language comments:
a. Section 2.3, step 3: suggested edit of sentence: ‘We recommend not to pellet nuclei down prior to freezing in order to prevent nuclei loss due to nuclei bursting and/or damage.
b. Section 3, note/warning: Separate into two sentences instead of just comma.
