# Supplementary material for: Optimised fluorescence-activated nuclei sorting for epigenomic analysis of cortical cell types
Source: PLoS One. 2026 Oct 6;21(10):e0359643. doi: 10.1371/journal.pone.0359643 (PMC13641707; doi:10.1371/journal.pone.0359643)
Supplement: Supplementary file 6 [file pone.0359643.s024.docx]

We thank the Reviewer for their positive comments on our manuscript and for confirming that we have addressed all major concerns raised during the previous round of review. We are grateful for their careful scrutiny of the revised manuscript and for identifying these remaining formatting issues. We have addressed each of the points raised, as detailed below and look forward to the successful publication of our manuscript.

**Reviewer #1:** The authors addressed all major concerns and comments with the revision.

There are a few minor formatting changes that should be addressed:

**1. Table 1: Alignment/formatting of Hoechst 33342 Species Human/Mouse into single line.**

**Response:** This formatting issue has been corrected.

**2. File S2: The Warning statement in section 3 should be updated to two sentences instead of a comma. Change to ‘Do not change the parameter settings between samples you wish to compare. If you do, you will need to re-record all samples using the changed parameters.’**

**Response:** We have made this change and republished the relevant protocols.io protocols. The corresponding URLs in the manuscript have also been updated.

**3. Formatting consistency of figure legends: Fig 2, and S9, S11 Fig legend panel letters are not bold but they are bold in other Figures (S1, S2, S3, S4, S5, S6, S7, S8). Furthermore, Line 563: (h&k) and (i&l) are not bolded but remainder of figure panels are.**

**Response:** We have standardised the formatting of the figure legends throughout the manuscript. We thank the Reviewer for identifying these inconsistencies.

**4. S2 Fig: There is an extra panel d) in the stained flow cytometry plots.**

**Response:** The extra panel label has been removed. We thank the Reviewer for pointing this out.

**5. S9 Fig: In the legend, there is indicated a and b panels, which is not clearly labeled in revised figure.**

**Response:** We have revised S9 Fig to provide clearer labelling of panels (a) and (b).

**6. S17 Fig legend, Line 714: Remove panel (A) indication.**

**Response:** The S17 Fig legend has been revised accordingly.
