# Supplementary material for: Optimised fluorescence-activated nuclei sorting for epigenomic analysis of cortical cell types
Source: PLoS One. 2026 Oct 6;21(10):e0359643. doi: 10.1371/journal.pone.0359643 (PMC13641707; doi:10.1371/journal.pone.0359643)
Supplement: Supplementary file 7 [file pone.0359643.s025.docx]

**Response to Reviewers' Comments**

We thank the Reviewer for their positive comments on our manuscript and for confirming that we have addressed all major concerns raised during the previous rounds of review. We are grateful for their careful assessment of the revised manuscript and for identifying the remaining formatting and labelling inconsistencies. We have addressed each of the points raised, as detailed below.

**Reviewer #1: The authors addressed all previous concerns. A final careful reading generated additional formatting and labeling inconsistencies that should be addressed prior to publication.**

**1. PlosOne suggests that euthanasia methods be indicated in the Ethics Statement. Currently, euthanasia methods are described in the text and methods section but missing from the ethics statement.**

**Response:** We thank the reviewer for highlighting this omission and apologise for the oversight. To address this, we have added the following statement describing the euthanasia method to the Ethics Statement:

*”Mice were sacrificed by cervical dislocation by trained personnel and frontal cortex tissue was dissected from 30 adult (1.5–12 months) wildtype C57BL/6J mice all in accordance with the UK Animals (Scientific Procedures) Act 1986 and with approval of the local Animal Welfare and Ethical Review Board (PPL P29FAC36A). Animals were monitored regularly throughout the study, maintained under standard husbandry conditions, and all efforts were made to minimise distress and suffering.”*

**2. Line 67: missing word, small amounts [of] post-mortem tissue**

**Response:** This formatting issue has been corrected.

**3. Line 240-241: Please revise these two sentences for clarity in regards to when DNA was extracted and what was treated with RNase A and Proteinase K (DNA or nuclei).**

**Response:** We have revised these sentences to clarify that the nuclei were treated with RNase A and Proteinase K prior to DNA extraction.

*“High molecular weight DNA was extracted from ~500,000 nuclei. Nuclei were treated with RNase A and Proteinase K prior to DNA extraction using a 1× volume of SPRI bead solution containing 20%PEG 8000 and 2.5M NaCl. DNA was subsequently purified using 0.1x Ampure XP beads and eluted in nuclease-free water.”*

**4. Line 327: Space insertion needed with new sentence. …(10,000-30,000 nuclei). Nuclei recovery…**

**Response:** This formatting issue has been corrected.

**5. Line 350 and Table S1 suggests that purified cell populations were checked for ENO2 expression as another marker of neurons. Data in S7a Fig does not present data for the ENO2 gene. Was this data meant to be incorporated? Otherwise, current confirmation of the cell population with RBFOX3 is sufficient and indication of confirmation with ENO2 can be removed.**

**Response:** Thank you for highlighting this. We did measure ENO2 expression, which showed the same cell-type-specific pattern as RBFOX3. However, due to formatting constraints in Fig. S7a and because the ENO2 data did not provide additional information beyond that provided by RBFOX3, these data were not included in the figure. To avoid confusion and improve consistency, we have now removed references to ENO2 from the manuscript and Table S1.

**6. Line 391: Remove period in order to associate (S10 Fig) indication with the appropriate sentence.**

**Response:** This formatting issue has been corrected.

**7. Please review carefully all flow plots for consistency of labeling and formatting across all figures, panels and files. Below are a few issues with the flow plots that were identified in this most recent careful read:**

**a. Figure 2: Panel C, labelling inconsistent with A and B (APC instead of APC-A)**

**Response:** This formatting issue has been corrected.

**b. S2 File, Figures 2e, 3a and b: Panel appears to have a sample specific name above the flow plot rather than a color/marker as with all other flow plots.**

**Response:** These Figures have been corrected.

**c. S1 Fig, S2 Fig, S3 Fig a&d, S6 Fig a,b,c and Figures in protocols S1, S2, S3: The first plot to identify proper events indicates the gated events as nuclei-sized particles in the text, but labels the gate as ‘cells’. I might suggest changing the gate title from ‘cells’ to ‘events’ or ‘particles’ to indicate the population that is used for further downstream gating as ‘cells’ is not appropriate.**

**Response:** We thank the reviewer for this comment. We agree that the events within this gate represent nuclei-sized particles rather than intact cells. To improve clarity and avoid referring to these events as cells, we have replaced the gate label “Cells” with “Cellular Material” in all relevant figures and protocol figures.

**d. S1 Fig, S2 Fig, S3 Fig b&e: SSC-H vs SSC-W is described in figure legend for doublet discrimination, but SSC-W versus SSC-A is shown in figure plots. Please correct figure legend text.**

**Response:** This formatting issue has been corrected.

e. Hoechst dye was used for labeling of nuclei and detected in the DAPI channel (S1 Fig, S2 Fig, S3 Fig c&f and S1-S3 files). It might be helpful to clarify that Hoechst is detected in the DAPI-labeled channel. Simply adding the info after Hoechst 33342 fluorescence (DAPI-A) would be sufficient to understand across the figure.

**Response:** We thank the reviewer for this helpful suggestion. We have clarified this throughout the relevant figures and protocol files by indicating that Hoechst 33342 fluorescence was detected in the DAPI channel (DAPI).

f. S3 Fig (g-j): Make labeling of cell populations in the flow plots more clear and consistent (similar with labeling of S1 Fig and S2 Fig). For instance, in S1 Fig g-l, the cell population markers are used to describe the gates rather than S3 Fig which uses the fluorophore (which could change as others adapt this protocol).

g. S3 Fig m: The colors that are indicated for microglia, neurons and oligodendrocytes do not match panel l or description of populations g-l. Potentially, a coloring strategy like S1 Fig may be helpful to match up the colors from labels of the same populations (Neurons and FITC Stained in the gating strategy would both be indicated in purple). This would help eliminate any confusion about different gating strategies for similar isolated cell populations.

**Response:** We thank the reviewer for these helpful suggestions. We have revised S3 Fig to make the labelling and colour scheme consistent with other figures. Specifically, the gates are now labelled according to the corresponding cell populations rather than the fluorophores, and the colours used to represent each cell population have been made consistent.

**8. S15 Fig: Labeling of figure should be added to indicate whether the lane is a library or a marker ladder lane.**

**Response:** We thank the reviewer for this suggestion. We presume that this comment refers to S14 Fig, which contains the TapeStation image. We have added labels to identify the four lanes shown on the left of the figure and have revised the figure legend to provide greater clarity.
