# Supplementary material for: Optimised fluorescence-activated nuclei sorting for epigenomic analysis of cortical cell types
Source: PLoS One. 2026 Oct 6;21(10):e0359643. doi: 10.1371/journal.pone.0359643 (PMC13641707; doi:10.1371/journal.pone.0359643)
Supplement: Peer review history

# Peer review: Decision Letter 0

**DOI:** 10.1371/journal.pone.0359643.r001

**Authors:** Nafisa Jadavji

25 May 2026

Dear Dr. Dempster,

Thank you for submitting your manuscript to PLOS ONE. After careful consideration, we feel that it has merit but does not fully meet PLOS ONE’s publication criteria as it currently stands. Therefore, we invite you to submit a revised version of the manuscript that addresses the points raised during the review process.

Please submit your revised manuscript by Jul 09 2026 11:59PM. If you will need more time than this to complete your revisions, please reply to this message or contact the journal office at plosone@plos.org. When you're ready to submit your revision, log on to https://www.editorialmanager.com/pone/ and select the 'Submissions Needing Revision' folder to locate your manuscript file.

- A letter that responds to each point raised by the academic editor and reviewer(s). You should upload this letter as a separate file labeled 'Response to Reviewers'.
- A marked-up copy of your manuscript that highlights changes made to the original version. You should upload this as a separate file labeled 'Revised Manuscript with Track Changes'.
- An unmarked version of your revised paper without tracked changes. You should upload this as a separate file labeled 'Manuscript'.

If you would like to make changes to your financial disclosure, please include your updated statement in your cover letter. Guidelines for resubmitting your figure files are available below the reviewer comments at the end of this letter.

If applicable, we recommend that you deposit your laboratory protocols in protocols.io to enhance the reproducibility of your results. Protocols.io assigns your protocol its own identifier (DOI) so that it can be cited independently in the future. For instructions see: https://journals.plos.org/plosone/s/submission-guidelines#loc-laboratory-protocols. Additionally, PLOS ONE offers an option for publishing peer-reviewed Lab Protocol articles, which describe protocols hosted on protocols.io. Read more information on sharing protocols at https://plos.org/protocols?utm_medium=editorial-email&utm_source=authorletters&utm_campaign=protocols.

As the corresponding author, your ORCID iD is verified in the submission system and will appear in the published article. PLOS supports the use of ORCID, and we encourage all coauthors to register for an ORCID iD and use it as well. Please encourage your coauthors to verify their ORCID iD within the submission system before final acceptance, as unverified ORCID iDs will not appear in the published article. *Only*  the individual author can complete the verification step; PLOS staff *cannot*  verify ORCID iDs on behalf of authors.

We look forward to receiving your revised manuscript.

Kind regards,

Nafisa M. Jadavji, PhD, MSc, BSc

Academic Editor

PLOS One

Journal requirements:

When submitting your revision, we need you to address these additional requirements.

1. Please ensure that your manuscript meets PLOS ONE's style requirements, including those for file naming. The PLOS ONE style templates can be found at

https://journals.plos.org/plosone/s/file?id=wjVg/PLOSOne_formatting_sample_main_body.pdf and

https://journals.plos.org/plosone/s/file?id=ba62/PLOSOne_formatting_sample_title_authors_affiliations.pdf

2. To comply with PLOS One submissions requirements, in your Methods section, please provide additional information regarding the experiments involving animals and ensure you have included details on (1) methods of sacrifice, (2) methods of anesthesia and/or analgesia, and (3) efforts to alleviate suffering.

3. Please include your tables as part of your main manuscript and remove the individual files. Please note that supplementary tables should remain uploaded as separate "supporting information" files.

4. Please include captions for your Supporting Information files at the end of your manuscript, and update any in-text citations to match accordingly. Please see our Supporting Information guidelines for more information: http://journals.plos.org/plosone/s/supporting-information.

If the reviewer comments include a recommendation to cite specific previously published works, please review and evaluate these publications to determine whether they are relevant and should be cited. There is no requirement to cite these works unless the editor has indicated otherwise.

Additional Editor Comments:

Your manuscript been reviewed by myself and another reviewer.

My comments to you are to describe whether there is a requirement for starting material quality. I think more trouble shooting tips should be included as well as representative results.

Please respond in detail to all comments made by myself and the review.

I look forward to reading your revised protocol manuscript.

[Note: HTML markup is below. Please do not edit.]

Reviewers' comments:

Reviewer's Responses to Questions

**Comments to the Author**

Reviewer #1: Yes

**********

2. Has the protocol been described in sufficient detail?

To answer this question, please click the link to protocols.io in the Materials and Methods section of the manuscript (if a link has been provided) or consult the step-by-step protocol in the Supporting Information files.

Reviewer #1: Yes

**********

3. Does the protocol describe a validated method?

Reviewer #1: Yes

**********

4. If the manuscript contains new data, have the authors made this data fully available?

The PLOS Data policy

Reviewer #1: Yes

**********

**5. Is the article presented in an intelligible fashion and written in standard English?**

Reviewer #1: Yes

**********

Reviewer #1: The article “Optimised fluorescence-activated nuclei sorting for epigenomic analysis of cortical cell types” by Chioza et al. describes a protocol (3 linked protocols) for isolation of individual neurons and glia cell types from mouse and human brain tissue using fluorescence-activated nuclei sorting (FANS) for downstream mutli-omics analysis. Compared to other protocols and studies that have focused on FANS isolation of a single neuronal cell type, this protocol describes optimization of parallel multi-omic profiling across multiple neural populations isolated from a small amount of post-mortem tissue. This protocol will be helpful to those investigating epigenomic changes in various cell types associated with disease using mouse and human tissue and make efficient use of tissue for data collection from multiple cell types, providing additional insight into brain disease pathogenesis which often affects multiple cell types. The authors provide data for each omics assay done to confirm appropriate population isolation and validation of the FANS technique.

Major suggestions for manuscript:

1. Line 101: Use the PLoSOne Protocol template preferred language.

2. General comment: In general, the flow cytometry data throughout S1-S3 files/manuscript are describing populations as positive and negative; however, without an unstained/IgG control to validate, it looks like in some cases, you might have high and low populations rather than a truly negative population. Could one example be included of an unstained control with the overlaid positively stained sample for an example of proper troubleshooting for a new marker?

3. Figure S7 and associated bulk RNA seq analysis: Do PC plots show clear separation of the distinct populations as is depicted by the clustering using the top variably expressed genes? Throughout, the cell type separation is validated by selection of the most variable genes/locations. It would be nice to have at least one PC analysis.

Minor Suggestions for manuscript:

1. Line 203: It mentions that two nuclei fractions were inspected, but specifies three (NeuN+, SOX10+ and total) populations. Please clarify if two or three and which populations were inspected.

2. Number mismatch for DNA methylation (ONT). It’s listed that genomic DNA was harvested from 50K-200K nuclei in Figure 1, but line 236 lists that genomic DNA was extracted from 500,000 nuclei for ONT sequencing and line 314 mentions 50K. Please double check nuclei amounts used for ONT or if there is a description missing.

3. Figure S9a; the labeling for DGFRB is covered/shortened.

4. One of the strengths of this protocol is that it gives a good depiction of how much data can be gathered from 300mg of human cortex tissue, which greatly helps in planning such studies. For mouse tissue, far less cells are capable of being isolated (lines 317-319). Do the authors have a good sense of the use of the number of nuclei needed in multiomics approaches and how best to plan use of fewer nuclei across multiple analyses with mice? Any insight would be valuable to those researchers using mice.

5. Line 471 suggests that a limitation is the cytometry platform and fluorophore capabilities. While this can be a limitation as multicolor experiments become more complicated and need additional troubleshooting, the current FANS protocol only uses 3 markers (and Hoescht), for a total of 4 colors. This is not the full capacity of the Aria III instrument they used. Is it really the cytometer that is limited or is more that the markers are limited in the available antibodies with flow cytometry-appropriate fluorophores? Please clarify.

6. Grammar/language comments:

a. Line 82: run-on sentence; Add a colon after ‘downstream genomic assays’.

b. Line 199, 264, 443: space before or reference

c. Line 203: parenthesis after total is superscript

d. Line 358: double wording ‘validates, confirms or both’

e. Line 388: Insert comma, ‘postnatal human, please refer to’

f. Line 447: comma inserted ‘and other cell types (46), our protocols are’

g. Line 457: comma insertion/ letter case change: ‘and astrocytes (LHX2), highlight that this strategy’

Minor suggestions to protocol S1-S3 files:

Protocol/ File S1:

1. Equipment missing: centrifuge, rotating mixer (File S1-S3).

2. Section 1.4, step 5: Suggested to resuspend in ‘-2mL’. Correct the negative volume.

3. Section 2.2 It’s mentioned that the flow rate can be increased in reduce sort ‘speed’. Are you actually referring to sort time, as increasing flow rate would increase the sort speed.

4. What is the interpretation for Figure 2: the FITC-A vs PE-A plot, it appears that NeuN+ cells are both PE+ and FITC+? Was compensation performed to address spectral overlap?

5. Grammar/language comments:

a. Section 2.3: Combine notes 1+2 about RNA Trizol and mixing tube so it’s clear what sample needs to be mixed. This happens again in File S2.

b. Section 2.3: Note 3: Repeated ‘at least’ for yield. Remove one.

Protocol/ File S2:

1. Section 1.3, step 6; no speed mentioned for centrifuge step.

2. Section 1.3, step 13: 1mL is repeated as final volume.

3. Section 1.4, step 2: instead of rotor, use rotator or rotating mixer.

4. There is a time for section 2, FANS as 5 minutes. This is misleading, as all the steps mentioned to start up the instrument and gating take much longer. Just remove a time as that would depend on sample.

5. Section 2 Note 1: There’s a reference made to ‘populations’ that should be present. What populations are being referred to here?

6. Figure 2 makes reference to letters for panels, but the panels are not labeled in the actual figure.

7. Section 2.2 mentions sort speed as reduced with a flow rate increase. See comment 3 under Protocol S1.

8. Figure 3b mentions poor sample separation. I suggest being more specific in that it is the population separation (Satb2+ versus Stab2-) that is poorer in this sample.

9. Grammar/language comments:

a. Section 1.3, step 1: ‘pour off any remaining supernatant’

b. There’s some strange spacing errors for section 1.3, step 2,3,9,12.

c. Section 1.3, step 9: insert comma, ‘If the sample was split, then pool together..’

d. Section 1.3, step 13: missing quotation around “Stained”

e. Section 2.3, numbers of steps are all repeated.

f. Note/Warning Section 3: Separate into two sentences instead of separating with comma.

Protocol/File S3:

1. Antibody and dyes (Table 5) are missing for this protocol file. Note that Table 5 is referenced in immunostaining step, section 1.4.

2. Section 1.2, step 5: Is there a suggestion for how much volume should be used per mg of tissue?

3. Section 1.3, step 7: Make clear that washing step instruction is a centrifugation.

4. Section 1.4, step 2: As with other files, use rotator or rotating mixer to explain equipment.

5. Grammar/language comments:

a. Section 2.3, step 3: suggested edit of sentence: ‘We recommend not to pellet nuclei down prior to freezing in order to prevent nuclei loss due to nuclei bursting and/or damage.

b. Section 3, note/warning: Separate into two sentences instead of just comma.

**********

what does this mean?). If published, this will include your full peer review and any attached files.

If you choose “no”, your identity will remain anonymous but your review may still be made public.

**Do you want your identity to be public for this peer review?**  For information about this choice, including consent withdrawal, please see our Privacy Policy

Reviewer #1: No

**********

[NOTE: If reviewer comments were submitted as an attachment file, they will be attached to this email and accessible via the submission site. Please log into your account, locate the manuscript record, and check for the action link "View Attachments". If this link does not appear, there are no attachment files.]

To ensure your figures meet our technical requirements, please review our figure guidelines: https://journals.plos.org/plosone/s/figures

You may also use PLOS’s free figure tool, NAAS, to help you prepare publication quality figures: https://journals.plos.org/plosone/s/figures#loc-tools-for-figure-preparation.

NAAS will assess whether your figures meet our technical requirements by comparing each figure against our figure specifications.

# Author comment: Author response to Decision Letter 1

**DOI:** 10.1371/journal.pone.0359643.r002

25 Jun 2026

Please also see the attached document named "Responses to Reviewers"

1. Journal requirements:

1. Please ensure that your manuscript meets PLOS ONE's style requirements, including those for file naming.

We have consulted the templates and made the necessary alterations to ensure that our manuscript meets the style requirements.

2. To comply with PLOS One submissions requirements, in your Methods section, please provide additional information regarding the experiments involving animals and ensure you have included details on (1) methods of sacrifice, (2) methods of anesthesia and/or analgesia, and (3) efforts to alleviate suffering.

The Methods section has been revised to include additional details regarding animal welfare considerations. All animals were humanely euthanised by cervical dislocation performed by trained personnel in accordance with Schedule 1 of the UK Animals (Scientific Procedures) Act 1986. No anaesthesia or analgesia was administered before euthanasia, as cervical dislocation is an approved Schedule 1 method of humane killing and was considered appropriate for the procedures undertaken. Animals were monitored regularly throughout the study, maintained under standard husbandry conditions, and all efforts were made to minimise distress and suffering. These details have now been added to the Methods section of the revised manuscript.

3. Please include your tables as part of your main manuscript and remove the individual files. Please note that supplementary tables should remain uploaded as separate "supporting information" files.

We have embedded Table 1 into the main document as requested.

4. Please include captions for your Supporting Information files at the end of your manuscript, and update any in-text citations to match accordingly. Please see our Supporting Information guidelines for more information: http://journals.plos.org/plosone/s/supporting-information.

This has been completed as requested – please see revised files.

2. Additional Editor Comments:

My comments to you are to describe whether there is a requirement for starting material quality. I think more trouble shooting tips should be included as well as representative results.

Thank you for these comments. We have added more troubleshooting tips on protocols.io where a detailed step-by-step protocol is available, including comments regarding starting material quality and advice on how to deal with nuclei clumping.

3.Reviewers' comments:

Major suggestions for manuscript:

1. Line 101: Use the PLoSOne Protocol template preferred language.

We have used the preferred language template to reference our step-by-step protocol on protocol.io.

2. General comment: In general, the flow cytometry data throughout S1-S3 files/manuscript are describing populations as positive and negative; however, without an unstained/IgG control to validate, it looks like in some cases, you might have high and low populations rather than a truly negative population. Could one example be included of an unstained control with the overlaid positively stained sample for an example of proper troubleshooting for a new marker?

We thank the reviewer for this helpful suggestion and have included unstained images for each of our FANS protocols in the revised Supplementary Figures S1-S3. These additions provide a clear reference for establishing fluorescence thresholds and demonstrate the distinction between background autofluorescence and true antibody-positive populations. We hope this will aid users in troubleshooting and adapting the protocols to additional markers or tissue sources, while also addressing the reviewer's point that some populations may otherwise be interpreted as high- and low-expressing populations rather than truly negative and positive populations.

3. Figure S7 and associated bulk RNA seq analysis: Do PC plots show clear separation of the distinct populations as is depicted by the clustering using the top variably expressed genes? Throughout, the cell type separation is validated by selection of the most variable genes/locations. It would be nice to have at least one PC analysis.

We thank the reviewer for this helpful suggestion and agree that an orthogonal dimensionality reduction approach provides additional support for the separation of the isolated cell populations beyond clustering based on highly variable genes. To address this point, we have now included a principal component analysis (PCA) plot in Figure S7C generated using the 5,000 most variably expressed genes. The PCA demonstrates clear separation between the two cell populations, providing an independent validation of the clustering results shown previously. We have updated Figure S7 and the corresponding text in the manuscript to highlight this additional validation of cell-type separation.

Minor Suggestions for manuscript:

1. Line 203: It mentions that two nuclei fractions were inspected, but specifies three (NeuN+, SOX10+ and total) populations. Please clarify if two or three and which populations were inspected.

Thank you for spotting this. We have corrected it to state “three”.

2. Number mismatch for DNA methylation (ONT). It’s listed that genomic DNA was harvested from 50K-200K nuclei in Figure 1, but line 236 lists that genomic DNA was extracted from 500,000 nuclei for ONT sequencing and line 314 mentions 50K. Please double check nuclei amounts used for ONT or if there is a description missing.

Thank you for highlighting this inconsistency. The different DNA methylation profiling approaches used in this study required different input amounts of genomic DNA and, consequently, different numbers of nuclei. We have revised Figure 1 to accurately reflect the nuclei input requirements for each method and have updated the relevant sections of the text to clarify which nuclei numbers correspond to each DNA methylation profiling approach.

3. Figure S9a; the labelling for DGFRB is covered/shortened.

The PDGFRB label was inadvertently obscured by a text box in the figure. This has now been corrected in the revised Figure S9A.

4. One of the strengths of this protocol is that it gives a good depiction of how much data can be gathered from 300mg of human cortex tissue, which greatly helps in planning such studies. For mouse tissue, far less cells are capable of being isolated (lines 317-319). Do the authors have a good sense of the use of the number of nuclei needed in multiomics approaches and how best to plan use of fewer nuclei across multiple analyses with mice? Any insight would be valuable to those researchers using mice.

We thank the reviewer for this valuable suggestion and agree that practical guidance around experimental planning is particularly useful for researchers working with mouse tissue, where nuclei yields are often substantially lower than those obtained from human cortex.

The number of nuclei required depends on the downstream application and the abundance of the target cell population. Based on our experience, for mouse cortex it is generally feasible to generate both DNA methylation data using the mouse DNA methylation array and ATAC-seq data from the more abundant NeuN+ neuronal population, with approximately 10,000–30,000 nuclei allocated to each assay. However, yields can decrease considerably when working with smaller brain regions or rarer cell populations, making it challenging to perform multiple downstream assays from a single sample. In such cases, careful experimental prioritisation is essential. Researchers may need to optimise nuclei isolation procedures, pool tissue samples where appropriate, or prioritise assays according to study objectives and expected effect sizes. We have added text to both the methods section and to the discussion to provide guidance on expected nuclei requirements and considerations for experimental planning in mouse studies.

5. Line 471 suggests that a limitation is the cytometry platform and fluorophore capabilities. While this can be a limitation as multicolor experiments become more complicated and need additional troubleshooting, the current FANS protocol only uses 3 markers (and Hoescht), for a total of 4 colors. This is not the full capacity of the Aria III instrument they used. Is it really the cytometer that is limited or is more that the markers are limited in the available antibodies with flow cytometry-appropriate fluorophores? Please clarify.

Although the FACS Aira III can support up to 5 lasers, our model is a 4-laser-configured instrument, and our approach utilises the spatial separation of the 4 laser spots to avoid fluorochrome leakage and negate the need for compensation. While it could be possible to add additional markers as each laser has multiple detectors, this would involve a more complicated experimental setup requiring compensation for fluorochromes detected using a shared excitatory laser, which could potentially reduce the sensitivity of detection. For this reason, we utilised a 4-colour approach, although we acknowledge that more markers could potentially be added using a more complex experimental design and make this point in the manuscript.

6. Grammar/language comments:

a. Line 82: run-on sentence; Add a colon after ‘downstream genomic assays’.

Resolved.

b. Line 199, 264, 443: space before or reference

Resolved.

c. Line 203: parenthesis after total is superscript

Resolved.

d. Line 358: double wording ‘validates, confirms or both’

Resolved.

e. Line 388: Insert comma, ‘postnatal human, please refer to’

Resolved.

f. Line 447: comma inserted ‘and other cell types (46), our protocols are’

Resolved.

g. Line 457: comma insertion/ letter case change: ‘and astrocytes (LHX2), highlight that this strategy’

Resolved.

Minor suggestions to protocol S1-S3 files:

We thank the reviewer for these detailed corrections below and have amended the three protocols accordingly and created revised versions on Protocols.io.

Protocol/ File S1:

1. Equipment missing: centrifuge, rotating mixer (File S1-S3).

2. Section 1.4, step 5: Suggested to resuspend in ‘-2mL’. Correct the negative volume.

3. Section 2.2 It’s mentioned that the flow rate can be increased in reduce sort ‘speed’. Are you actually referring to sort time, as increasing flow rate would increase the sort speed.

4. What is the interpretation for Figure 2: the FITC-A vs PE-A plot, it appears that NeuN+ cells are both PE+ and FITC+? Was compensation performed to address spectral overlap?

5. Grammar/language comments:

a. Section 2.3: Combine notes 1+2 about RNA Trizol and mixing tube so it’s clear what sample needs to be mixed. This happens again in File S2.

b. Section 2.3: Note 3: Repeated ‘at least’ for yield. Remove one.

Protocol/ File S2:

1. Section 1.3, step 6; no speed mentioned for centrifuge step.

2. Section 1.3, step 13: 1mL is repeated as final volume.

3. Section 1.4, step 2: instead of rotor, use rotator or rotating mixer.

4. There is a time for section 2, FANS as 5 minutes. This is misleading, as all the steps mentioned to start up the instrument and gating take much longer. Just remove a time as that would depend on sample.

5. Section 2 Note 1: There’s a reference made to ‘populations’ that should be present. What populations are being referred to here?

6. Figure 2 makes reference to letters for panels, but the panels are not labeled in the actual figure.

7. Section 2.2 mentions sort speed as reduced with a flow rate increase. See comment 3 under Protocol S1.

8. Figure 3b mentions poor sample separation. I suggest being more specific in that it is the population separation (Satb2+ versus Stab2-) that is poorer in this sample.

9. Grammar/language comments:

a. Section 1.3, step 1: ‘pour off any remaining supernatant’

b. There’s some strange spacing errors for section 1.3, step 2,3,9,12.

c. Section 1.3, step 9: insert comma, ‘If the sample was split, then pool together..’

d. Section 1.3, step 13: missing quotation around “Stained”

e. Section 2.3, numbers of steps are all repeated.

f. Note/Warning Section 3: Separate into two sentences instead of separating with comma.

Protocol/File S3:

1. Antibody and dyes (Table 5) are missing for this protocol file. Note that Table 5 is referenced in immunostaining step, section 1.4.

2. Section 1.2, step 5: Is there a suggestion for how much volume should be used per mg of tissue?

3. Section 1.3, step 7: Make clear that washing step instruction is a centrifugation.

4. Section 1.4, step 2: As with other files, use rotator or rotating mixer to explain equipment.

5. Grammar/language comments:

a. Section 2.3, step 3: suggested edit of sentence: ‘We recommend not to pellet nuclei down prior to freezing in order to prevent nuclei loss due to nuclei bursting and/or damage.

b. Section 3, note/warning: Separate into two sentences instead of just comma.

Attachment Submitted filename: fans_paper_response_reviewerFINALv2.docx

# Peer review: Decision Letter 1

**DOI:** 10.1371/journal.pone.0359643.r003

**Authors:** Nafisa Jadavji

21 Jul 2026

Dear Dr. Dempster,

Thank you for submitting your manuscript to PLOS ONE. After careful consideration, we feel that it has merit but does not fully meet PLOS ONE’s publication criteria as it currently stands. Therefore, we invite you to submit a revised version of the manuscript that addresses the points raised during the review process.

Please submit your revised manuscript by Sep 04 2026 11:59PM. If you will need more time than this to complete your revisions, please reply to this message or contact the journal office at plosone@plos.org. When you're ready to submit your revision, log on to https://www.editorialmanager.com/pone/ and select the 'Submissions Needing Revision' folder to locate your manuscript file.

- A letter that responds to each point raised by the academic editor and reviewer(s). You should upload this letter as a separate file labeled 'Response to Reviewers'.
- A marked-up copy of your manuscript that highlights changes made to the original version. You should upload this as a separate file labeled 'Revised Manuscript with Track Changes'.
- An unmarked version of your revised paper without tracked changes. You should upload this as a separate file labeled 'Manuscript'.

If you would like to make changes to your financial disclosure, please include your updated statement in your cover letter. Guidelines for resubmitting your figure files are available below the reviewer comments at the end of this letter.

If applicable, we recommend that you deposit your laboratory protocols in protocols.io to enhance the reproducibility of your results. Protocols.io assigns your protocol its own identifier (DOI) so that it can be cited independently in the future. For instructions see: https://journals.plos.org/plosone/s/submission-guidelines#loc-laboratory-protocols. Additionally, PLOS ONE offers an option for publishing peer-reviewed Lab Protocol articles, which describe protocols hosted on protocols.io. Read more information on sharing protocols at https://plos.org/protocols?utm_medium=editorial-email&utm_source=authorletters&utm_campaign=protocols.

As the corresponding author, your ORCID iD is verified in the submission system and will appear in the published article. PLOS supports the use of ORCID, and we encourage all coauthors to register for an ORCID iD and use it as well. Please encourage your coauthors to verify their ORCID iD within the submission system before final acceptance, as unverified ORCID iDs will not appear in the published article. *Only*  the individual author can complete the verification step; PLOS staff *cannot*  verify ORCID iDs on behalf of authors.

We look forward to receiving your revised manuscript.

Kind regards,

Nafisa M. Jadavji, PhD, MSc, BSc

Academic Editor

PLOS One

Journal Requirements:

If the reviewer comments include a recommendation to cite specific previously published works, please review and evaluate these publications to determine whether they are relevant and should be cited. There is no requirement to cite these works unless the editor has indicated otherwise.

Please review your reference list to ensure that it is complete and correct. If you have cited papers that have been retracted, please include the rationale for doing so in the manuscript text, or remove these references and replace them with relevant current references. Any changes to the reference list should be mentioned in the rebuttal letter that accompanies your revised manuscript. If you need to cite a retracted article, indicate the article’s retracted status in the References list and also include a citation and full reference for the retraction notice.

[Note: HTML markup is below. Please do not edit.]

Reviewers' comments:

Reviewer's Responses to Questions

**Comments to the Author**

Reviewer #1: Yes

**********

2. Has the protocol been described in sufficient detail?

To answer this question, please click the link to protocols.io in the Materials and Methods section of the manuscript (if a link has been provided) or consult the step-by-step protocol in the Supporting Information files.

Reviewer #1: Yes

**********

3. Does the protocol describe a validated method?

Reviewer #1: Yes

**********

4. If the manuscript contains new data, have the authors made this data fully available?

The PLOS Data policy

Reviewer #1: Yes

**********

**5. Is the article presented in an intelligible fashion and written in standard English?**

Reviewer #1: Yes

**********

Reviewer #1: The authors addressed all major concerns and comments with the revision.

There are a few minor formatting changes that should be addressed:

1. Table 1: Alignment/formatting of Hoeschst 33342 Species Human/Mouse into single line.

2. File S2: The Warning statement in section 3 should be updated to two sentences instead of a comma. Change to ‘Do not change the parameter settings between samples you wish to compare. If you do, you will need to re-record all samples using the changed parameters.’

3. Formatting consistency of figure legends: Fig 2, and S9,S11 Fig legend panel letters are not bold but they are bold in other Figures (S1, S2, S3, S4, S5, S6, S7, S8). Furthermore, Line 563: (h&k) and (i&l) are not bolded but remainder of figure panels are.

4. S2 Fig: There is an extra panel d) in the stained flow cytometry plots.

5. S9 Fig: In the legend, there is indicated a and b panels, which is not clearly labeled in revised figure.

6. S17 Fig legend, Line 714: Remove panel (A) indication

**********

what does this mean?). If published, this will include your full peer review and any attached files.

If you choose “no”, your identity will remain anonymous but your review may still be made public.

**Do you want your identity to be public for this peer review?**  For information about this choice, including consent withdrawal, please see our Privacy Policy

Reviewer #1: No

**********

[NOTE: If reviewer comments were submitted as an attachment file, they will be attached to this email and accessible via the submission site. Please log into your account, locate the manuscript record, and check for the action link "View Attachments". If this link does not appear, there are no attachment files.]

To ensure your figures meet our technical requirements, please review our figure guidelines: https://journals.plos.org/plosone/s/figures

You may also use PLOS’s free figure tool, NAAS, to help you prepare publication quality figures: https://journals.plos.org/plosone/s/figures#loc-tools-for-figure-preparation.

NAAS will assess whether your figures meet our technical requirements by comparing each figure against our figure specifications.

# Author comment: Author response to Decision Letter 2

**DOI:** 10.1371/journal.pone.0359643.r004

10 Aug 2026

We thank the Reviewer for their positive comments on our manuscript and for confirming that we have addressed all major concerns raised during the previous round of review. We are grateful for their careful scrutiny of the revised manuscript and for identifying these remaining formatting issues. We have addressed each of the points raised, as detailed below and look forward to the successful publication of our manuscript.

Reviewer #1: The authors addressed all major concerns and comments with the revision.

There are a few minor formatting changes that should be addressed:

1. Table 1: Alignment/formatting of Hoechst 33342 Species Human/Mouse into single line.

Response: This formatting issue has been corrected.

2. File S2: The Warning statement in section 3 should be updated to two sentences instead of a comma. Change to ‘Do not change the parameter settings between samples you wish to compare. If you do, you will need to re-record all samples using the changed parameters.’

Response: We have made this change and republished the relevant protocols.io protocols. The corresponding URLs in the manuscript have also been updated.

3. Formatting consistency of figure legends: Fig 2, and S9, S11 Fig legend panel letters are not bold but they are bold in other Figures (S1, S2, S3, S4, S5, S6, S7, S8). Furthermore, Line 563: (h&k) and (i&l) are not bolded but remainder of figure panels are.

Response: We have standardised the formatting of the figure legends throughout the manuscript. We thank the Reviewer for identifying these inconsistencies.

4. S2 Fig: There is an extra panel d) in the stained flow cytometry plots.

Response: The extra panel label has been removed. We thank the Reviewer for pointing this out.

5. S9 Fig: In the legend, there is indicated a and b panels, which is not clearly labeled in revised figure.

Response: We have revised S9 Fig to provide clearer labelling of panels (a) and (b).

6. S17 Fig legend, Line 714: Remove panel (A) indication.

Response: The S17 Fig legend has been revised accordingly.

Attachment Submitted filename: Fans paper rebuttal no2.docx

# Peer review: Decision Letter 2

**DOI:** 10.1371/journal.pone.0359643.r005

**Authors:** Nafisa Jadavji

3 Sep 2026

Dear Dr. Dempster,

Thank you for submitting your manuscript to PLOS One. After careful consideration, we feel that it has merit but does not fully meet PLOS One’s publication criteria as it currently stands. Therefore, we invite you to submit a revised version of the manuscript that addresses the points raised during the review process.

**Dear Authors,** **Some minor revisions are required.** **Sincerely,** **Nafisa**

==============================

Please submit your revised manuscript by Oct 18 2026 11:59PM. If you will need more time than this to complete your revisions, please reply to this message or contact the journal office at plosone@plos.org. When you're ready to submit your revision, log on to https://www.editorialmanager.com/pone/ and select the 'Submissions Needing Revision' folder to locate your manuscript file.

- A letter that responds to each point raised by the academic editor and reviewer(s). You should upload this letter as a separate file labeled 'Response to Reviewers'.
- A marked-up copy of your manuscript that highlights changes made to the original version. You should upload this as a separate file labeled 'Revised Manuscript with Track Changes'.
- An unmarked version of your revised paper without tracked changes. You should upload this as a separate file labeled 'Manuscript'.

If you would like to make changes to your financial disclosure, please include your updated statement in your cover letter. Guidelines for resubmitting your figure files are available below the reviewer comments at the end of this letter.

If applicable, we recommend that you deposit your laboratory protocols in protocols.io to enhance the reproducibility of your results. Protocols.io assigns your protocol its own identifier (DOI) so that it can be cited independently in the future. For instructions see: https://journals.plos.org/plosone/s/submission-guidelines#loc-laboratory-protocols. Additionally, PLOS One offers an option for publishing peer-reviewed Lab Protocol articles, which describe protocols hosted on protocols.io. Read more information on sharing protocols at https://plos.org/protocols?utm_medium=editorial-email&utm_source=authorletters&utm_campaign=protocols.

As the corresponding author, your ORCID iD is verified in the submission system and will appear in the published article. PLOS supports the use of ORCID, and we encourage all coauthors to register for an ORCID iD and use it as well. Please encourage your coauthors to verify their ORCID iD within the submission system before final acceptance, as unverified ORCID iDs will not appear in the published article. *Only*  the individual author can complete the verification step; PLOS staff *cannot*  verify ORCID iDs on behalf of authors.

We look forward to receiving your revised manuscript.

Kind regards,

Nafisa M. Jadavji, PhD, MSc, BSc

Academic Editor

PLOS One

Journal Requirements:

If the reviewer comments include a recommendation to cite specific previously published works, please review and evaluate these publications to determine whether they are relevant and should be cited. There is no requirement to cite these works unless the editor has indicated otherwise.

Please review your reference list to ensure that it is complete and correct. If you have cited papers that have been retracted, please include the rationale for doing so in the manuscript text, or remove these references and replace them with relevant current references. Any changes to the reference list should be mentioned in the rebuttal letter that accompanies your revised manuscript. If you need to cite a retracted article, indicate the article’s retracted status in the References list and also include a citation and full reference for the retraction notice.

[Note: HTML markup is below. Please do not edit.]

Reviewers' comments:

Reviewer's Responses to Questions

**Comments to the Author**

Reviewer #1: Yes

**********

2. Has the protocol been described in sufficient detail?

To answer this question, please click the link to protocols.io in the Materials and Methods section of the manuscript (if a link has been provided) or consult the step-by-step protocol in the Supporting Information files.

Reviewer #1: Yes

**********

3. Does the protocol describe a validated method?

Reviewer #1: Yes

**********

4. If the manuscript contains new data, have the authors made this data fully available?

The PLOS Data policy

Reviewer #1: Yes

**********

**5. Is the article presented in an intelligible fashion and written in standard English?**

Reviewer #1: Yes

**********

Reviewer #1: The authors addressed all previous concerns. A final careful reading generated additional formatting and labeling inconsistencies that should be addressed prior to publication.

1. PlosOne suggests that euthanasia methods be indicated in the Ethics Statement. Currently, euthanasia methods are described in the text and methods section but missing from the ethics statement.

2. Line 67: missing word, small amounts [of] post-mortem tissue

3. Line 240-241: Please revise these two sentences for clarity in regards to when DNA was extracted and what was treated with RNase A and Proteinase K (DNA or nuclei).

4. Line 327: Space insertion needed with new sentence. …(10,000-30,000 nuclei). Nuclei recovery…

5. Line 350 and Table S1 suggests that purified cell populations were checked for ENO2 expression as another marker of neurons. Data in S7a Fig does not present data for the ENO2 gene. Was this data meant to be incorporated? Otherwise, current confirmation of the cell population with RBFOX3 is sufficient and indication of confirmation with ENO2 can be removed.

6. Line 391: Remove period in order to associate (S10 Fig) indication with the appropriate sentence.

7. Please review carefully all flow plots for consistency of labeling and formatting across all figures, panels and files. Below are a few issues with the flow plots that were identified in this most recent careful read:

a. Figure 2: Panel C, labeling inconsistent with A and B (APC instead of APC-A)

b. S2 File, Figures 2e, 3a and b: Panel appears to have a sample specific name above the flow plot rather than a color/marker as with all other flow plots.

c. S1 Fig, S2 Fig, S3 Fig a&d, S6 Fig a,b,c and Figures in protocols S1, S2, S3: The first plot to identify proper events indicates the gated events as nuclei-sized particles in the text, but labels the gate as ‘cells’. I might suggest changing the gate title from ‘cells’ to ‘events’ or ‘particles’ to indicate the population that is used for further downstream gating as ‘cells’ is not appropriate.

d. S1 Fig, S2 Fig, S3 Fig b&e: SSC-H vs SSC-W is described in figure legend for doublet discrimination, but SSC-W versus SSC-A is shown in figure plots. Please correct figure legend text.

e. Hoechst dye was used for labeling of nuclei and detected in the DAPI channel (S1 Fig, S2 Fig, S3 Fig c&f and S1-S3 files). It might be helpful to clarify that Hoechst is detected in the DAPI-labeled channel. Simply adding the info after Hoechst 33342 fluorescence (DAPI-A) would be sufficient to understand across the figure.

f. S3 Fig (g-j): Make labeling of cell populations in the flow plots more clear and consistent (similar with labeling of S1 Fig and S2 Fig). For instance, in S1 Fig g-l, the cell population markers are used to describe the gates rather than S3 Fig which uses the fluorophore (which could change as others adapt this protocol).

g. S3 Fig m: The colors that are indicated for microglia, neurons and oligodendrocytes do not match panel l or description of populations g-l. Potentially, a coloring strategy like S1 Fig may be helpful to match up the colors from labels of the same populations (Neurons and FITC Stained in the gating strategy would both be indicated in purple). This would help eliminate any confusion about different gating strategies for similar isolated cell populations.

8. S15 Fig: Labeling of figure should be added to indicate whether the lane is a library or a marker ladder lane.

**********

what does this mean?). If published, this will include your full peer review and any attached files.

If you choose “no”, your identity will remain anonymous but your review may still be made public.

**Do you want your identity to be public for this peer review?**  For information about this choice, including consent withdrawal, please see our Privacy Policy

Reviewer #1: No

**********

[NOTE: If reviewer comments were submitted as an attachment file, they will be attached to this email and accessible via the submission site. Please log into your account, locate the manuscript record, and check for the action link "View Attachments". If this link does not appear, there are no attachment files.]

To ensure your figures meet our technical requirements, please review our figure guidelines: https://journals.plos.org/plosone/s/figures

You may also use PLOS’s free figure tool, NAAS, to help you prepare publication quality figures: https://journals.plos.org/plosone/s/figures#loc-tools-for-figure-preparation.

NAAS will assess whether your figures meet our technical requirements by comparing each figure against our figure specifications.

# Author comment: Author response to Decision Letter 3

**DOI:** 10.1371/journal.pone.0359643.r006

10 Sep 2026

We thank the Reviewer for their positive comments on our manuscript and for confirming that we have addressed all major concerns raised during the previous rounds of review. We are grateful for their careful assessment of the revised manuscript and for identifying the remaining formatting and labelling inconsistencies. We have addressed each of the points raised, as detailed below.

Reviewer #1: The authors addressed all previous concerns. A final careful reading generated additional formatting and labeling inconsistencies that should be addressed prior to publication.

1. PlosOne suggests that euthanasia methods be indicated in the Ethics Statement. Currently, euthanasia methods are described in the text and methods section but missing from the ethics statement.

Response: We thank the reviewer for highlighting this omission and apologise for the oversight. To address this, we have added the following statement describing the euthanasia method to the Ethics Statement:

”Mice were sacrificed by cervical dislocation by trained personnel and frontal cortex tissue was dissected from 30 adult (1.5–12 months) wildtype C57BL/6J mice all in accordance with the UK Animals (Scientific Procedures) Act 1986 and with approval of the local Animal Welfare and Ethical Review Board (PPL P29FAC36A). Animals were monitored regularly throughout the study, maintained under standard husbandry conditions, and all efforts were made to minimise distress and suffering.”

2. Line 67: missing word, small amounts [of] post-mortem tissue

Response: This formatting issue has been corrected.

3. Line 240-241: Please revise these two sentences for clarity in regards to when DNA was extracted and what was treated with RNase A and Proteinase K (DNA or nuclei).

Response: We have revised these sentences to clarify that the nuclei were treated with RNase A and Proteinase K prior to DNA extraction.

“High molecular weight DNA was extracted from ~500,000 nuclei. Nuclei were treated with RNase A and Proteinase K prior to DNA extraction using a 1× volume of SPRI bead solution containing 20%PEG 8000 and 2.5M NaCl. DNA was subsequently purified using 0.1x Ampure XP beads and eluted in nuclease-free water.”

4. Line 327: Space insertion needed with new sentence. …(10,000-30,000 nuclei). Nuclei recovery…

Response: This formatting issue has been corrected.

5. Line 350 and Table S1 suggests that purified cell populations were checked for ENO2 expression as another marker of neurons. Data in S7a Fig does not present data for the ENO2 gene. Was this data meant to be incorporated? Otherwise, current confirmation of the cell population with RBFOX3 is sufficient and indication of confirmation with ENO2 can be removed.

Response: Thank you for highlighting this. We did measure ENO2 expression, which showed the same cell-type-specific pattern as RBFOX3. However, due to formatting constraints in Fig. S7a and because the ENO2 data did not provide additional information beyond that provided by RBFOX3, these data were not included in the figure. To avoid confusion and improve consistency, we have now removed references to ENO2 from the manuscript and Table S1.

6. Line 391: Remove period in order to associate (S10 Fig) indication with the appropriate sentence.

Response: This formatting issue has been corrected.

7. Please review carefully all flow plots for consistency of labeling and formatting across all figures, panels and files. Below are a few issues with the flow plots that were identified in this most recent careful read:

a. Figure 2: Panel C, labelling inconsistent with A and B (APC instead of APC-A)

Response: This formatting issue has been corrected.

b. S2 File, Figures 2e, 3a and b: Panel appears to have a sample specific name above the flow plot rather than a color/marker as with all other flow plots.

Response: These Figures have been corrected.

c. S1 Fig, S2 Fig, S3 Fig a&d, S6 Fig a,b,c and Figures in protocols S1, S2, S3: The first plot to identify proper events indicates the gated events as nuclei-sized particles in the text, but labels the gate as ‘cells’. I might suggest changing the gate title from ‘cells’ to ‘events’ or ‘particles’ to indicate the population that is used for further downstream gating as ‘cells’ is not appropriate.

Response: We thank the reviewer for this comment. We agree that the events within this gate represent nuclei-sized particles rather than intact cells. To improve clarity and avoid referring to these events as cells, we have replaced the gate label “Cells” with “Cellular Material” in all relevant figures and protocol figures.

d. S1 Fig, S2 Fig, S3 Fig b&e: SSC-H vs SSC-W is described in figure legend for doublet discrimination, but SSC-W versus SSC-A is shown in figure plots. Please correct figure legend text.

Response: This formatting issue has been corrected.

e. Hoechst dye was used for labeling of nuclei and detected in the DAPI channel (S1 Fig, S2 Fig, S3 Fig c&f and S1-S3 files). It might be helpful to clarify that Hoechst is detected in the DAPI-labeled channel. Simply adding the info after Hoechst 33342 fluorescence (DAPI-A) would be sufficient to understand across the figure.

Response: We thank the reviewer for this helpful suggestion. We have clarified this throughout the relevant figures and protocol files by indicating that Hoechst 33342 fluorescence was detected in the DAPI channel (DAPI).

f. S3 Fig (g-j): Make labeling of cell populations in the flow plots more clear and consistent (similar with labeling of S1 Fig and S2 Fig). For instance, in S1 Fig g-l, the cell population markers are used to describe the gates rather than S3 Fig which uses the fluorophore (which could change as others adapt this protocol).

g. S3 Fig m: The colors that are indicated for microglia, neurons and oligodendrocytes do not match panel l or description of populations g-l. Potentially, a coloring strategy like S1 Fig may be helpful to match up the colors from labels of the same populations (Neurons and FITC Stained in the gating strategy would both be indicated in purple). This would help eliminate any confusion about different gating strategies for similar isolated cell populations.

Response: We thank the reviewer for these helpful suggestions. We have revised S3 Fig to make the labelling and colour scheme consistent with other figures. Specifically, the gates are now labelled according to the corresponding cell populations rather than the fluorophores, and the colours used to represent each cell population have been made consistent.

8. S15 Fig: Labeling of figure should be added to indicate whether the lane is a library or a marker ladder lane.

Response: We thank the reviewer for this suggestion. We presume that this comment refers to S14 Fig, which contains the TapeStation image. We have added labels to identify the four lanes shown on the left of the figure and have revised the figure legend to provide greater clarity.

Attachment Submitted filename: PlosOne_fansprotocol_ResponsetoReviewerV3.docx

# Editor report: Decision Letter 3

**DOI:** 10.1371/journal.pone.0359643.r007

**Authors:** Nafisa Jadavji

16 Sep 2026

Optimised fluorescence-activated nuclei sorting for epigenomic analysis of cortical cell types

PONE-D-26-02584R3

Dear Dr. Dempster,

We’re pleased to inform you that your manuscript has been judged scientifically suitable for publication and will be formally accepted for publication once it meets all outstanding technical requirements.

Within one week, you’ll receive an e-mail detailing the required amendments. When these have been addressed, you’ll receive a formal acceptance letter and your manuscript will be scheduled for publication.

An invoice will be generated when your article is formally accepted. Please note, if your institution has a publishing partnership with PLOS and your article meets the relevant criteria, all or part of your publication costs will be covered. Please make sure your user information is up-to-date by logging into Editorial Manager at Editorial Manager® and clicking the ‘Update My Information' link at the top of the page. For questions related to billing, please contact billing support.

If your institution or institutions have a press office, please notify them about your upcoming paper to help maximize its impact. If they’ll be preparing press materials, please inform our press team as soon as possible -- no later than 48 hours after receiving the formal acceptance. Your manuscript will remain under strict press embargo until 2 pm Eastern Time on the date of publication. For more information, please contact onepress@plos.org.

Kind regards,

Nafisa M. Jadavji, PhD, MSc, BSc

Academic Editor

PLOS One

Additional Editor Comments (optional):

Reviewers' comments:

# Editor report: Acceptance letter

**DOI:** 10.1371/journal.pone.0359643.r008

**Authors:** Nafisa Jadavji

PONE-D-26-02584R3

PLOS One

Dear Dr. Dempster,

I'm pleased to inform you that your manuscript has been deemed suitable for publication in PLOS One. Congratulations! Your manuscript is now being handed over to our production team.

At this stage, our production department will prepare your paper for publication. This includes ensuring the following:

* All references, tables, and figures are properly cited

* All relevant supporting information is included in the manuscript submission,

* There are no issues that prevent the paper from being properly typeset

You will receive further instructions from the production team, including instructions on how to review your proof when it is ready. Please keep in mind that we are working through a large volume of accepted articles, so please give us a few days to review your paper and let you know the next and final steps.

Lastly, if your institution or institutions have a press office, please let them know about your upcoming paper now to help maximize its impact. If they'll be preparing press materials, please inform our press team within the next 48 hours. Your manuscript will remain under strict press embargo until 2 pm Eastern Time on the date of publication. For more information, please contact onepress@plos.org.

You will receive an invoice from PLOS for your publication fee after your manuscript has reached the completed accept phase. If you receive an email requesting payment before acceptance or for any other service, this may be a phishing scheme. Learn how to identify phishing emails and protect your accounts at https://explore.plos.org/phishing.

If we can help with anything else, please email us at customercare@plos.org.

Thank you for submitting your work to PLOS One and supporting open access.

Kind regards,

PLOS One Editorial Office Staff

on behalf of

Dr. Nafisa M. Jadavji

Academic Editor

PLOS One
